# Artemisinin resistance in the malaria parasite, Plasmodium falciparum, originates from its initial transcriptional response

**DOI:** 10.1038/s42003-022-03215-0

**Published:** 2022-03-28

**Authors:** Lei Zhu, Rob W. van der Pluijm, Michal Kucharski, Sourav Nayak, Jaishree Tripathi, Nicholas J. White, Nicholas P. J. Day, Abul Faiz, Aung Pyae Phyo, Chanaki Amaratunga, Dysoley Lek, Elizabeth A. Ashley, François Nosten, Frank Smithuis, Hagai Ginsburg, Lorenz von Seidlein, Khin Lin, Mallika Imwong, Kesinee Chotivanich, Mayfong Mayxay, Mehul Dhorda, Hoang Chau Nguyen, Thuy Nhien Thanh Nguyen, Olivo Miotto, Paul N. Newton, Podjanee Jittamala, Rupam Tripura, Sasithon Pukrittayakamee, Thomas J. Peto, Tran Tinh Hien, Arjen M. Dondorp, Zbynek Bozdech

**Affiliations:** 1 School of Biological Sciences, Nanyang Technological University, Singapore, Singapore; 2 Mahidol-Oxford Tropical Medicine Research Unit, Faculty of Tropical Medicine, Mahidol University, Bangkok, Thailand; 3 Centre for Tropical Medicine and Global Health, Nuffield Department of Medicine, University of Oxford, Oxford, UK; 4 Center of Tropical Medicine and Travel Medicine, Department of Infectious Diseases, Academic Medical Center, University of Amsterdam, Amsterdam, The Netherlands; 5 Ramu Upazila Health Complex, Cox’s Bazar, Bangladesh; 6 Malaria Research Group and Dev Care Foundation, Dhaka, Bangladesh; 7 Myanmar Oxford Clinical Research Unit, Yangon, Myanmar; 8 Laboratory of Malaria and Vector Research, National Institute of Allergy and Infectious Diseases, National Institutes of Health, Rockville, MD USA; 9 National Centre for Parasitology, Entomology and Malaria Control, Phnom Penh, Cambodia; 10 Lao-Oxford-Mahosot Hospital Wellcome Trust Research Unit, Vientiane, Laos; 11 Shoklo Malaria Research Unit, Mahidol-Oxford Tropical Medicine Research Unit, Faculty of Tropical Medicine, Mahidol University, Mae Sot, Thailand; 12 The Alexander Silberman Institute of Life Science, Hebrew University of Jerusalem, Jerusalem, Israel; 13 Department of Medical Research, Pyin Oo Lwin, Myanmar; 14 Department of Molecular Tropical Medicine and Genetics, Faculty of Tropical Medicine, Mahidol University, Bangkok, Thailand; 15 Institute of Research and Education Development (IRED), University of Health Sciences, Ministry of Health, Vientiane, Laos; 16 WorldWide Antimalarial Resistance Network – Asia-Pacific Regional Centre, Bangkok, Thailand; 17 Oxford University Clinical Research Unit, Hospital for Tropical Diseases, Ho Chi Minh City, Vietnam; 18 Medical Research Council (MRC) Centre for Genomics and Global Health, University of Oxford, Oxford, UK; 19 Wellcome Trust Sanger Institute, Hinxton, Cambridge UK; 20 Department of Clinical Tropical Medicine, Faculty of Tropical Medicine, Mahidol University, Bangkok, Thailand; 21 The Royal Society of Thailand, Dusit, Bangkok, Thailand

**Keywords:** Parasite genomics, Predictive markers, Evolutionary genetics

## Abstract

The emergence and spread of artemisinin-resistant *Plasmodium falciparum*, first in the Greater Mekong Subregion (GMS), and now in East Africa, is a major threat to global malaria elimination ambitions. To investigate the artemisinin resistance mechanism, transcriptome analysis was conducted of 577 *P. falciparum* isolates collected in the GMS between 2016–2018. A specific artemisinin resistance-associated transcriptional profile was identified that involves a broad but discrete set of biological functions related to proteotoxic stress, host cytoplasm remodelling, and REDOX metabolism. The artemisinin resistance-associated transcriptional profile evolved from initial transcriptional responses of susceptible parasites to artemisinin. The genetic basis for this adapted response is likely to be complex.

## Abstract (web summary)

Transcriptomic analysis of isolates from the malaria parasite (*Plasmodium falciparum*) in the Greater Mekong Subregion of Southeast Asia identifies gene expression patterns that are correlated with resistance to a common anti-malaria drug, artemisinin.

## Introduction

Artemisinin-based combination therapies (ACTs) have been critical to the success in reducing the global burden of falciparum malaria between 2000 and 2015^1^. Loss of these drugs to resistance would be a disaster. Historically the Greater Mekong Subregion (GMS) has been the origin of antimalarial drug resistance in *P. falciparum*. In recent years the emergence and spread of artemisinin resistance and, subsequently, partner drug resistance has led to high failure rates of ACTs in several parts of the GMS^2,3^. Recently artemisinin resistance has arisen independently in East Africa (parts of Rwanda and Uganda)^4^. The phenotypic manifestation of artemisinin-resistant *P. falciparum* infections in vivo is slowed parasite clearance after treatment with artesunate. The slow clearance phenotype, defined by a parasite clearance half-life (PC½) > 5 h, is attributed to loss of sensitivity of *P. falciparum* to artemisinins during the early stage of the intraerythrocytic developmental cycle (IDC), ring stage^5,6^ and is causally associated with nonsynonymous mutations in the propeller region of the *P. falciparum kelch 13* gene (*PfK13*)^7,8^. Artemisinin resistance was first reported in western Cambodia, Pailin province in 2009. Initially, over 20 different *PfK13* mutations were associated with the slow parasite clearance phenotype^8–10^. However, since 2013 the soft sweeps resulting in multiple emergences of *PfK13* mutations in the eastern part of the GMS were largely replaced by a selective sweep of a haplotype bearing a single nonsynonymous *PfK13* SNP (C580Y)^11^. This single lineage of artemisinin-resistant *P. falciparum* spread and expanded through western and northern Cambodia, northeastern Thailand and southern Vietnam, and Lao PDR^8,12,13^. This was soon joined with molecular markers associated with resistance to the ACT partner drug piperaquine. PfK13-propeller domain mutations in artemisinin-resistant parasite lineages have also emerged independently and spread through Myanmar and western Thailand^14^. Moreover, PfK13-propeller domain mutations have been reported in Northern India^15^, and more recent foci include independent emergence in Papua New Guinea^16^, Rwanda^4^, Ethiopia^17^, and other parts of sub-Saharan Africa^18^.

The molecular mechanism by which the *PfK13* mutations confer artemisinin resistance is a subject of intense research using in vitro and in vivo models (reviewed in^19–23^). Collectively, these studies have proposed the involvement of multiple cellular and metabolic processes in artemisinin resistance including haemoglobin degradation, proteotoxic/unfolded protein stress response, vesicular biogenesis as well as oxidative stress response and mitochondrial functions. Translational suppression mediated by phosphorylation of eIF2α, linked to cell quiescence and slowing of the IDC can also confer artemisinin resistance in vitro^24^. Missense or loss-of-function alleles of other genes were also shown to contribute to artemisinin resistance in vitro. These include coronin^25^, falcipain2a/b^26^, ubiquitin hydrolase (*pcubp1*)^27^, and μ-subunit of the AP2 vesicular trafficking complex (*pcap2*)^27^. Thus, it appears that artemisinin resistance is mediated by a multifaceted mechanism that results from a concerted action of several metabolic and cellular factors. These may act in different, not mutually exclusive, combinations^28^. Undoubtedly these mechanisms drive artemisinin resistance of *P. falciparum* in in vitro conditions in which the parasites are supplied with superfluous amounts of nutrients, kept at uniform temperature, are not targeted by the host’s immune system and other ambient stresses exerted by the host’s environment^29^. The question now is what are the roles of each of these identified components of artemisinin resistance in natural infections, in vivo.

To investigate this, several genome-wide association studies (GWAS) were conducted. These identified large regions on chromosome 10 and 13, and 14^30,31^, and subsequently seven nonsynonymous SNPs associated with *PfK13* SNPs^32^. A concurrent longitudinal study of the GMS parasites collected between 2001 and 2014 suggested that additional genes might be associated with artemisinin resistance including an additional *kelch* protein on chromosome 10^33^. Collectively, these studies detected genes that could be loosely linked with the artemisinin resistance-implicated biological functions, however, no experimental evidence of their role in the artemisinin resistance clinical phenotype has been reported so far.

Transcriptome-wide association analysis (TWAS) is currently emerging as the method of choice for identifying causative genetic variations of complex traits in a wide range of biological systems ranging from plants^34^ to human^35–37^. This is based on the wealth of GWAS studies showing that the vast majority of genetic polymorphisms associated with complex genetic traits (such as genetic diseases) lay within the noncoding regions^38^. These are typically affecting DNA regulatory sequences and thus gene expression through which the phenotype is manifested^39^. This likely also applies to *P. falciparum* as suggested by our earlier study investigating expression quantitative trait loci (eQTL) in the TRACI parasite isolates^40^. We then carried out transcriptome analyses of the *P. falciparum* parasites from the Tracking Resistance to Artemisinin Collaboration (TRACI) study conducted in the GMS countries between 2011 and 2013^8,41^. This showed that artemisinin resistance is associated with broad transcriptional changes of many genes, some of which may be linked with inductions of the unfolded protein response (UPR) and with a general deceleration of the IDC. Here we present a transcriptome analysis of parasite isolates from a more recent cohort of *P. falciparum* natural infections collected in the GMS between 2016 and 2018; 5–7 years after the initial TRACI study^8^, and after the recent selective sweep of *PfK13* C580Y^2^. We identified a spectrum of transcriptionally correlated genes that likely contribute to artemisinin resistance via their altered transcriptional levels. We termed this the artemisinin resistance-associated transcriptional profile (ARTP) and provide evidence that its constitutive expression may have evolved from the initial transcriptional responses of sensitive *P. falciparum* parasites to the artemisinins.

## Results

### Transcriptome of the *P. falciparum* population in the GMS 2016–2018

**Fig. 1:**
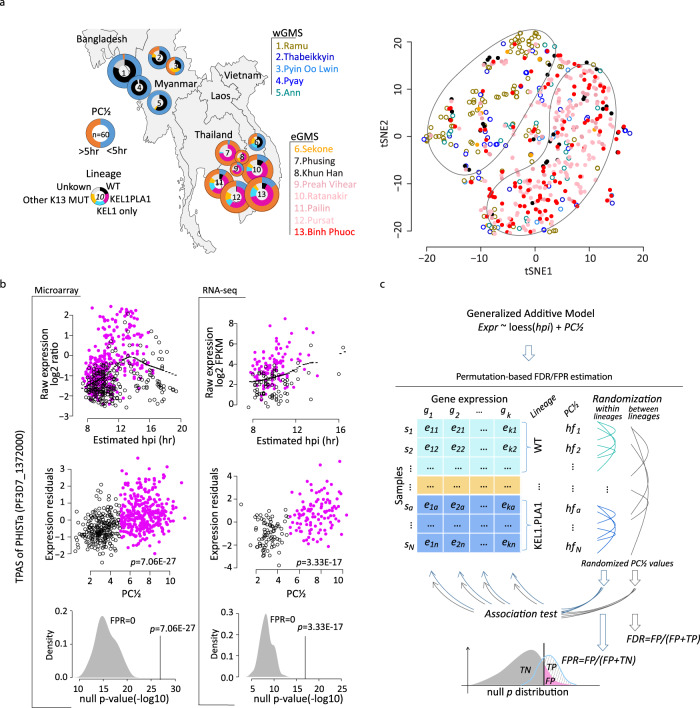
Transcriptome of TRACII *P. falciparum* parasite population and a schematic illustration of the TPAS methodology. **a** Geographic distribution of all samples used in this study. Pie charts represent the proportion of slow (PC½ > 5 h) and fast (PC½ < 5 h) clearing parasites, or lineages in categories based on *PfK13* mutations and plasmepsin II/III copy numbers (WT, KEL1 only, KEL1PLA1, other *PfK13* MUT and unknown). On the right, 577 samples before drug treatment are plotted to display two main clusters formed by the eGMS or wGMS parasites geographical distribution using t-SNE algorithm based on the top 2-12 PCs. **b** TPAS explanation using an example gene of PHISTa (PF3D7_1372000). The expression-hpi/age relationship is shown as the raw expression level (log2 ratio) plotted against the estimated hpi for the microarray data on the left (577 samples) and RNA-seq data on the right (188 samples) with purple dots representing resistant parasites (PC½ > 5 h), black circles for susceptible parasites (PC½ < 5 h) and black dotted lines for the loess curve using all dots. The expression-resistance relationship is represented by the expression residuals plotted against the PC½ for each data set. The density plots at the bottom represent 100 times permutation result within lineages for the FPR calculation. **c** Workflow of FDR and FPR estimation for the TPAS. The null *p* distribution was built using permutated resistance status (PC½ values) across parasite samples within lineages for FPR estimation and between lineages for multiple testing correction.

The main purpose of this study was to identify specific genes, presumably acting in concordance with the *PfK13* mutations, whose expression activity mediates/contributes to the physiological state that enables the parasite cell to withstand the parasiticidal effects of artemisinins. We conducted transcriptome analysis of *P. falciparum* parasites derived from the blood of patients with uncomplicated *P. falciparum* infections in 13 field sites across 6 GMS countries (Fig. 1a and Supplementary Table 1). These samples were collected during a large clinical treatment trial (TRACII) carried out from 2016 to 2018 that also characterized the spread of artemisinin resistance in the GMS^2^. We isolated total *P. falciparum* RNA from the patients’ blood samples and performed both DNA microarray and Next Generation Sequencing (RNA-seq) analysis^42^. Overall, we analyzed 577 samples collected at study enrolment to characterize the baseline transcriptomic profiles (baseline, ^(bl)^0 h sample set) (Supplementary Fig. 1a). For 459 (of the 577) patients, samples were also collected 6 h after administering an ACT to characterize the transcriptional response of *P. falciparum* parasites to the artemisinin in vivo (transcriptional response, ^(tr)^6 h sample set) (Supplementary Fig. 1b). While the DNA microarray was used to generate the transcriptomes for all collected samples, there were sufficient levels of parasite mRNA to perform RNA-seq-based transcriptome analyses for 188 and 159 of the ^(bl)^0 h and ^(tr)^6 h samples respectively^42^ (Supplementary Fig. 1c, d).

From the ^(bl)^0 h sample set, we established the distribution of the *P. falciparum* life cycle developmental stages in the peripheral blood including the asexual IDC stages expressed as hours post invasion (hpi) and the fractions of sexual stages (gametocytes)^40^ (Supplementary Fig. 2 and Supplementary Data 1). The ^(bl)^0 h sample set represented synchronized ring stage parasites between 6 and 20 hpi, with ≤ 5% of the overall parasitemia corresponding to gametocytes. Principle component analysis (PCA) of the ^(bl)^0 h transcriptome revealed parasite hpi composed the major transcriptome difference across samples as it is strongly correlated (Spearman *rho* = 0.87) with the first principal component (PC1) accounting for up to 32% of the overall transcriptome variation (Supplementary Fig. 3a). A significant non-linear relationship was observed between the hpi and expression (*p* < 0.001) of most of the studied genes measured by DNA microarrays (70%) and RNA-seq (62%, Supplementary Fig. 4). In contrast to the PC1, none of the subsequent top 2 to 12 PCs (each contributing > 1% of the overall transcriptome variation) correlated with any epidemiological variables (i.e. patient sex, age and time of collection etc.) and molecular biology parameters (i.e., RNA yield, quality assessments etc.) suggesting that the generated dataset was largely unaffected by the methodological approach or epidemiology aspects of the study (Supplementary Fig. 3a). The t-distributed stochastic neighbour embedding (t-SNE) method using the PC2-12 revealed two major parasite groups which separated according to the geographic regions: western GMS (wGMS) and eastern GMS (eGMS) (Fig. 1a). This grouping pattern also corresponds to the prevalence of artemisinin resistance in the eGMS that is demarcated by the expansion of the *P. falciparum* lineage carrying the *PfK13* C580Y mutation (named KEL1 lineage or PfPailin) of which 76% also carried multiple copies of plasmepsin II (KEL1PLA1, Fig. 1a)^2^. *PfK13* wild type parasites from the eGMS did not exhibit any strong association with either group, which further strengthens this model (Supplementary Fig. 3a). Altogether these results suggest that the selective sweep of the PfPailin or KEL1PLA1 lineage(s) in the eGMS over the last 5–7 years^43^ is mirrored by transcriptional convergence with the eGMS parasites that is distinct from the wGMS. This transcriptome pattern is presumably either contributing to the resistance mechanism and/or alleviating a fitness cost to support its selection.

### Transcriptome-phenotype association study of artemisinin resistance

In human genetics, TWAS typically combine GWAS of a studied phenotype on one side and independent datasets linking DNA elements with transcription on the other^36,37^. By the virtue of being a unicellular organism, in *P. falciparum*, we can correlate transcriptional differences with the studied phenotype (here artemisinin resistance) directly; the approach we term Transcriptome-Phenotype Association Studies (TPAS) (see “Methods”). First, we conducted TPAS analysis on the ^(bl)^0 h samples in order to identify genes whose steady state mRNA levels correlated with the level of artemisinin resistance represented by PC½. For this we applied a generalized additive model to relate each gene’s expression to the PC½ with a loess function along hpi (example in Fig. 1b). In this model, the expression residuals reflect the relative mRNA abundance unaffected by hpi and thus can be directly correlated to PC½^2^. To control for multiple testing in correlating between mRNA levels and PC½ values for the whole transcriptome, we calculated the false discovery rate (FDR) for each gene based on 1000 permutations (Fig. 1c). The expression-resistance association could also represent an expression-lineage relationship since the artemisinin resistance status is confounded with K13 lineages that 77% of the susceptible (PC½ < 5 h) samples were from the WT parasites in wGMS and 95% of the resistant (PC½ < 5 h) samples were from the K13 mutant in eGMS (Fig. 1a). Due to this homogeneity, expression-resistance associations will have a lineage effect if we use the entire data set for TPAS analysis or lose power if we test only within sub geographical region (w/eGMS). To overcome this, we estimated false positive rates (FPR) for each gene to control the type I error by repeating the analysis 100 times with randomly generated permutations (Fig. 1c), a method commonly used in human genetics studies^44,45^. In each permutation, the lineage structure was maintained and PC½ values were randomized amongst the parasites within each lineage. Subsequently FPR was calculated based on the null p distribution reflecting the probability of expression-resistance association caused by expression-lineage relationship. FPR < 0.05 (95% confidence) was applied to define robust expression-resistance associations beyond parasite lineage effect. By this we aimed to eliminate expression-lineage associations and identified transcripts whose expression levels are associated strictly with the PC½. One of the clearest examples was PHISTa (PF3D7_1372000) which displayed a strong association with artemisinin resistance at *p* = 7.06E-27 (FDR = 0) with FPR = 0 (Fig. 1b, for more examples see Supplementary Fig. 5).

**Fig. 2:**
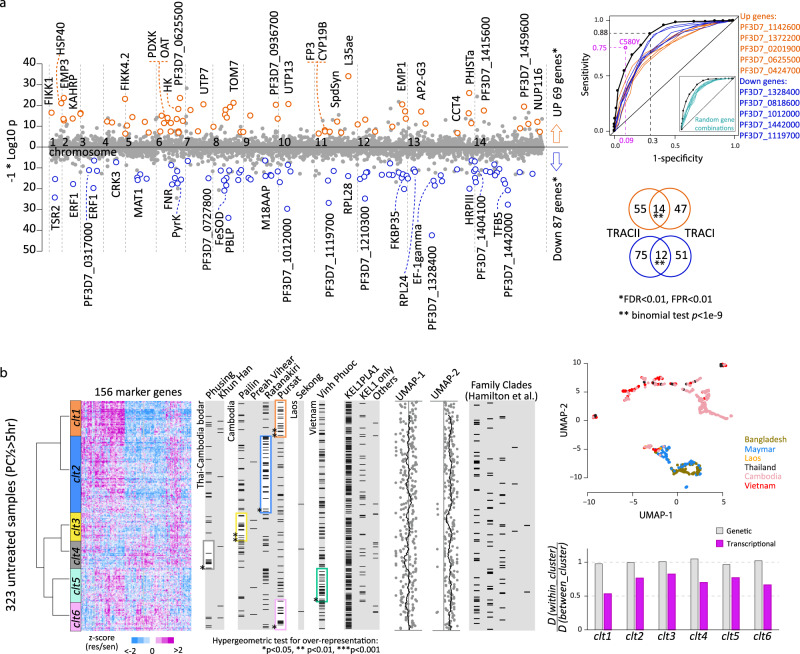
Transcriptional resistance markers. **a** The scatter plot represents all studied genes along the genomic coordinates on the *X*-axis with their resistance-association *p*-values displayed as -log10 *p* on the Y-axis. Genes passing the threshold (FDR < 0.05, FPR < 0.05) are highlighted in 69 orange circles (upregulation) and 87 blue circles (downregulation). The ROC plot shows the ability to distinguish resistant and susceptible parasites in TRACII for 5 upregulated genes (orange) and 5 downregulated genes (blue) with the best performance. Their gene IDs are shown on the side from top to bottom for the line from left corner to the centre of the plot. The black line with dots represents the distinguishing ability of the combination of all the 156 genes which was calculated as the sum up of expression values of the 69 upregulations with a subtraction of the sum up of the 87 downregulations. It is compared to the roc of randomly selected gene set on the bottom right (see “Methods”). The Venn diagrams show the overlap of TPAS results between the TRACII and TRACI studies. **b** A heatmap represents the transcriptional profiles clustering for 323 eGMS ^(bl)^0 h samples showing prolonged PC½( > 5 h) based on the 156 resistance markers. The colour (purple to blue) indicates the level of differential expression (upregulation to downregulation) in the resistant parasites. The left dendrogram represents Ward’s clustering result and the colour bars represent the 6 clusters obtained by clustering tree cutting. On the right, samples in each column (marked by black bars) are categorized by their respective sites or lineages, or population structure. Frames mark the overrepresentations of categorized samples in the corresponding clusters. *p < 0.05, **p < 0.01 and ***p < 0.001. The scatter plot represents the population structure of the whole GMS samples, and the bar plot represents the ratio of distance between within-clusters individuals and between-clusters individuals (see “Methods”).

Next, we applied the above TPAS method to the DNA microarray-derived data of 577 ^(bl)^0 h samples and RNA-seq-derived data of 188 ^(bl)^0 hr samples separately. We observed good correlation (Spearman *rho* = 0.68) between the TPAS results obtained from these two platforms (Supplementary Fig. 6). Merging the two results, we identified 69 upregulated and 87 downregulated transcripts whose levels were significantly associated with artemisinin resistance (FDR < 0.05, corresponding to *p* < 1e−10 in microarray, *p* < 1e−6 in RNA-seq, note: these criteria were subsequently used to define the ARTP, Fig. 2a). Out of these, 60S ribosomal protein gene, L35ae (PF3D7_1142600), PHISTa (PF3D7_1372000) erythrocyte membrane protein PfEMP3 (PF3D7_0219000), and PF3D7_1328400, BEM46 (PF3D7_0818600), and PF3D7_1012000 showed the strongest correlations with PC½ for up- and downregulated genes, respectively. Overall, both the upregulated and downregulated genes contribute to a broad but defined set of biological functions previously linked with artemisinin resistance such as protein and REDOX metabolism, digestive vacuole- and mitochondrial-linked biological functions, host cell remodelling etc. (Supplementary Data 2, also see below). When compared to the transcripts identified by the same approach in parasites collected during TRACI (between 2011–2013)^41^, we observed a significant overlap (binomial test *p* < 1e−9) in upregulated and downregulated genes, including the PHISTa, KAHRP, FIKK, ATG5, and FKBP35 (for full list see Supplementary Data 2). For further analysis, we term the transcriptional variations of the 156 selected genes (69 upregulated and 87 downregulated) the artemisinin resistance-associated transcriptional profile (ARTP) and investigate further its biological relevance for the putative artemisinin resistance mechanism. We also show that the individual components of the ARTP can be used to predict/diagnose artemisinin resistance either individually or in combinations (Fig. 2a). Specifically, the receiver operating characteristic (ROC) curve combining the 156 ARTP genes was able to distinguish between resistant and susceptible parasites in the studied dataset with a high level of sensitivity (0.88) and specificity (0.7) which is close to the performance of the C580Y mutation (sensitivity = 0.75 and specificity = 0.91).

### Artemisinin resistance-associated transcriptional profile

Next, we aimed to investigate the utility of the ARTP as a marker of the spread and evolution of artemisinin resistance in the GMS and possibly beyond. Hence, we conducted a clustering analysis to the 323 resistant parasite samples (PC½ > 5 h). Specifically, the Ward’s method was applied for clustering based on the Euclidean distance matrix of similarity of the ARTP formed by the 156 transcript levels in each of the ^(bl)^0 h samples. This approach identified six clusters that displayed a distinct geographical segregation (hypergeometric test *p* < 0.05) (Fig. 2b). It yielded two main clades with high relatedness between cluster 1 (Pursat) and 2 (Ratanakiri) on one side and the rests of the sites in clusters 3–6 including, Phusing (4) and Binh Phuoc (5) and Pailin (3). The majority of the 323 samples were from KEL1PLA1 lineage (216/323, 67%) with the remainder made up of 36 (11%) from KEL1, 19 (6%) from other lineages, and 52 (17%) from unknown lineages. No lineage bias was observed with any cluster. To further investigate the impact of population-driven genetic factors, population structure of the entire GMS samples was analyzed by PLINK^46^ and visualized using UMAP^47^. The UMAP-2 clearly distinguishes the eGMS parasites from the wGMS and UMAP-1 mainly reflects the genetic differences among parasites within each region (Fig. 2b). Either the UMAP-1 or UMAP-2 lack significant correlation to the individual clusters that suggest the genetic differences differentiating the eGMS from wGMS or the population substructure is not a significant confounding factor for the ARTP differential expression. To strengthen the validity of this observation we compared the genetic differences to the transcriptional differences between clusters. The ratio of average distance was calculated between within-cluster individuals and between-clusters individuals genetically and transcriptionally separately (see “Methods”). Here the genetic distance ratios are consistently ~1 across the cluster 1–6 which contrasts the transcriptional distance ratios that were 0.7 in average, ranging from 0.5 to 0.8 (Fig. 2b). This further indicates that the individual clusters are defined by their transcriptional characteristics but indistinguishable genetically. Moreover, no correlation was observed between the ARTP transcriptional clusters and previously reported subpopulations which reflect the founder population effect of the KEL1PLA1 parasites expansion^43^ (Fig. 2b). Taken together, the results from the UMAP analysis, the genetic/transcriptional distance ratio, and the funder subpopulation associations suggest that the ARTP represents transcriptional re-tuning of at least 156 genes that is largely independent of background genetic differences. Although, the ARTP seems to function mainly in conjunction with the nonsynonymous *PfK13* SNP, it is feasible to speculate that this expression profile could drive (at least to some degree) resistance to artemisinins even in an *PfK13*-independat manner.

### In vivo transcriptional response of *P. falciparum* to ACTs

**Fig. 3:**
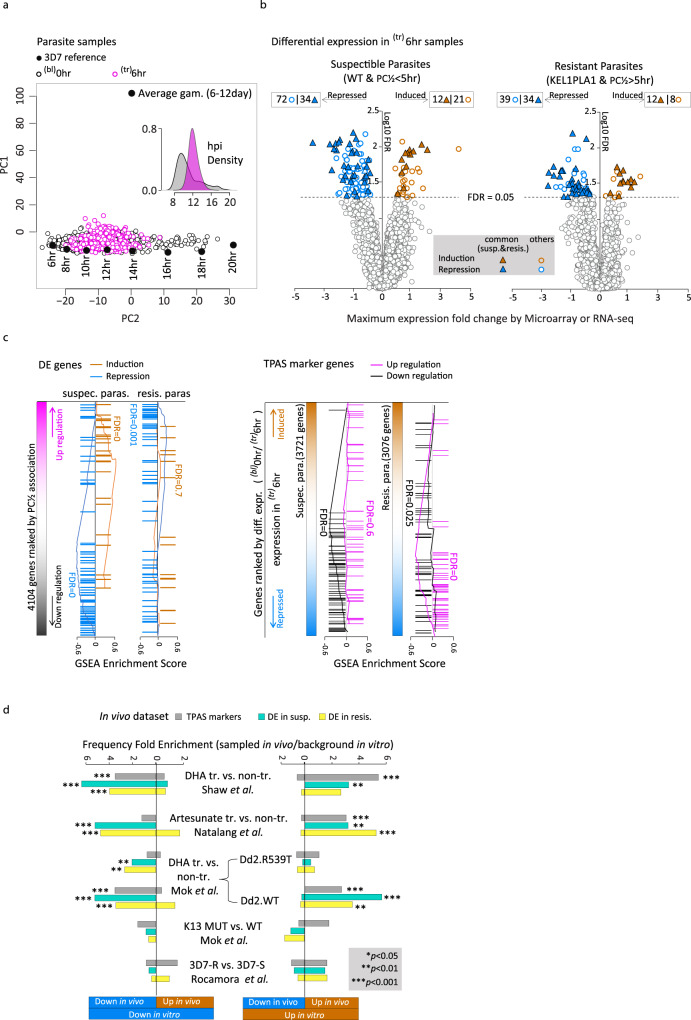
In vivo transcriptional response to artemisinins. **a** The principal components space of PC1 vs. PC2 was constructed by the PCA on reference transcriptomes of the laboratory strain 3D7 at ring stage and gametocyte stages (average of the day 5th–12th). It was used to visualize transcriptome differences driven by parasite age/hpi or developmental stage (asexual/sexual). The 577 ^(bl)^0 h (black circles) and 459 ^(tr)^6 h (purple circles) samples are projected onto this space to show their age differences. The density plot represents the estimated hpi distribution for ^(bl)^0 hr (grey) and ^tr)^6 hr (purple) parasites. **b** Volcano plot represents each gene’s association *p* value of differential expression against the average expression fold change between the ^(tr)^6 h and ^(bl)^0 h parasites for the susceptible group (left, WT with PC½ < 5 h) and the resistant group (right, KELPLA1 with PC½ > 5 h) respectively. **c** Genes differentially expressed as upregulation/induction (orange) and downregulation/repression (blue) are marked along the rank of their association to PC½ (from TPAS). Markers associated with PC½ positively (purple) or negatively (black) are marked along the rank of their differential expression levels (from **b**). GSEA was applied to estimate the FDR for ranking bias to either side of upregulation/induction or downregulation/repression. **d** Bar plots represent significant overlaps between our in vivo study and other independent in vitro studies, In vivo: baseline TPAS analysis (^(bl)^0 h, 156 resistant markers, grey); post-treatment differentially expressed genes (^(tr)^6 h/^(bl)^0 h) in susceptible (turquoise) and resistant parasites (yellow) group, In vitro: transcriptional response to DHA treatment in the K1 rings^48^; Artesunate treatment in the FCR3 strain^49^; DHA treatment in the Dd2 R539T or WT stain^50^; differential expression at the baseline level between the *PfK13* MUT and WT stains^50^ as well as that between lab-derived ART-resistant and ART-sensitive 3D7 ring parasites^51^. Stars mark intersections having > 3 genes with hypergeometric test *p* < 0.05. .

To complement the baseline TPAS, we evaluated the ^(tr)^6 h sample set to assess transcriptional responses of *P. falciparum* exposed to ACT in vivo. The ^(tr)^6 h parasites exhibited a tighter distribution of the IDC stages compared to those in the ^(bl)^0 h, falling between 8 and 14 hpi with a median ~12 hpi, which might reflect the more rapid loss of mature ring stage parasites because of their higher sensitivity to artemisinins (Fig. 3a and Supplementary Fig. 2) (authors’ note). There was also a reduction (2–5%) in the fraction of gametocytes. Comparative transcriptome analysis between the ^(bl)^0 h and ^(tr)^6 h was conducted to identify transcriptional responses for the resistant (KEL/PLA1 with PC½ > 5 h) and susceptible (WT with PC½ < 5 h) parasites separately (for details see “Methods”). In total, we identified 20 and 73 genes that were induced or repressed, respectively, after 6 h in vivo treatment in the KEL1PLA1/resistant parasites. Similarly, 33 and 106 genes were induced or repressed, respectively, in the WT/susceptible parasites (FDR < 0.05, corresponding *p* < 1e–14) (Fig. 3b). Highly consistent results were observed between the pairwise and non-pairwise analysis with Pearson Correlation Coefficient of the average expression change (resistant/susceptible) being 0.99 (Supplementary Fig. 7). Given the marked differences in the hpi distributions between the ^(bl)^0 h and ^(tr)^6 h parasite sets (Fig. 3a), the non-pairwise analysis provides additional confidence for the differential genes expression, reaching beyond the individual strain differences. Interestingly, the susceptible and resistant parasites exhibited distinct transcriptional responses to artemisinin with a minimal (albeit statistically significant) overlap including 12 and 34 commonly induced and repressed genes, respectively (Supplementary Data 2). Out of these, six genes were also downregulated in the baseline ^(bl)^0 h sample set while the only upregulated gene in both sample sets was PHISTa (PF3D7_1372000). There was a skewed distribution of the ^(tr)^6 hr induced and repressed genes in the WT/susceptible parasites along the distribution of the transcriptional associations of the ^(bl)^0 h parasites (Fig. 3c). Specifically, the 33 drug-induced genes were ranked significantly towards upregulation in ^(bl)^0 h and the 106 drug-repressed genes towards downregulation (FDR = 0). Contrastingly, the ^(tr)^6 h induced genes in the PfPailin (KEL1PLA1)/resistant parasites show no association with the ^(bl)^0 h baseline ARTP but the ^(tr)^6 h repressed genes exhibit a moderate overlap with upregulated genes in the ^(bl)^0 h baseline ARTP (Fig. 3c). This suggests that the transcriptional response of *P. falciparum* to 6 h artemisinin exposure in vivo is related to the ARTP which could be its precursor (see “Discussion”).

There were highly significant gene-by-gene overlaps between the in vivo results and several in vitro studies of transcriptional responses of *P. falciparum* to artemisinin(s) (Fig. 3d and Supplementary Table 2). This applies to the ^(bl)^0 h TPAS as well as the in vivo transcriptional response of ^(tr)^6 h parasites. There are good agreements between these results and earlier studies of in vitro transcriptional response of *P. falciparum* K1 strain exposed to dihydroartemisinin (DHA) for 1 h^48^, the FCR3 strain exposed to artesunate for 3 hours^49^ and the recently reported transcriptional responses of the Dd2^wt^ strain exposed to DHA for 6 hours^50^. This is consistent with our observation showing that the basal level ARTP overlaps well with transcriptional responses of the susceptible parasites (Fig. 3c). We also observed a limited overlap with our previously derived in vitro *P. falciparum* parasites with ring stage-specific artemisinin resistance^51^ suggesting that additional mechanisms conferring artemisinin resistance exist and could rise in vivo in the future.

## Discussion

This study identified at least 156 genes whose altered transcription may contribute significantly to artemisinin resistance. Artemisinin resistance involves a complex array of processes that have been selected to counter the parasiticidal effect of the drug^20–23^. These processes occur early in the asexual cycle and attenuate ring stage parasite killing. We found marked overlaps between the ARTP and the genes involved in the transcriptional response to the direct action of artemisinin observed in vivo (Fig. 3a, b) and/or in vitro^48–51^. This suggests that the mechanisms contributing to artemisinin resistance have arisen (at least in part) from the initial transcriptional response of *P. falciparum* parasites to the direct effect of the drug in which the artemisinin induced transcriptional changes became constitutive. Indeed, there are many overlaps on the gene-by-gene level between gene expression changes in the baseline ARTP and genes induced/suppressed by artemisinins in vivo and in vitro (Supplementary Table 2). In particular, we identified many determinants of protein metabolism including translation, folding, and degradation that were also found to be a part of *P. falciparum* response to artesunate^49^, and DHA of both susceptible and resistant parasite lines in vitro^48,50,51^. Notable examples include gene encoding 60S ribosomal subunits (L24), and elongation factors EF-1-gamma whose proteins products were found to be direct artemisinin targets in the parasite cells^52,53^. Related to this, we observed significant transcriptional changes for several determinants of protein turnover and protein folding from which DnaJ proteins and the T-complex-protein 1 subunit are likely to be direct protein targets of the artemisinin drugs (Supplementary Table 2). We also found transcriptional suppression of factors involved in REDOX functionalities, some of which are related to mitochondrial functions recently shown to play a key role in artemisinin resistance in vitro^50^. There was marked transcriptional activity of genes involved in biosynthetic pathways including pyridoxine/polyamine and purine/pyrimidine synthesis and glycolysis. Interestingly several enzymes encoded by these transcripts were also found to be inhibited by artemisinin directly including ornithine aminotransferase (OAT), spermidine synthase, pyruvate kinase, and hexokinase^52,53^. This also applies to the *P. falciparum* pyridoxal kinase, PDXK, whose mammalian orthologue, was shown to interact directly and being inhibited by artesunate^54^. Finally, the ARTP contains strong baseline-level upregulations of several transcripts encoding proteins exported to the host erythrocyte. These are implicated in host cell remodelling and/or host-parasite interactions that are paralleled by drug-induced transcriptional responses (Supplementary Table 2) (see below). Taken together, these observations support a model in which the initially adaptive transcriptional response became constitutively expressed as a result of drug selection, predisposing the parasite to withstand the drug’s parasiticidal effect.

**Fig. 4:**
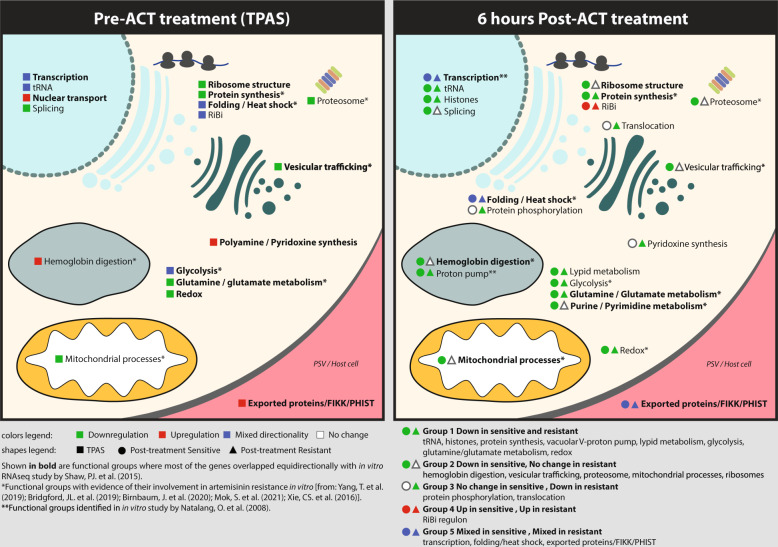
Functional assignment of resistance markers. Functional assignments of transcriptional artemisinin resistance markers derived from TPAS analysis at baseline-level (^(bl)^0 h) and that of genes repressed/induced after 6 h of ACT treatment (^(tr)^6 h). Colours represent transcriptome directionality for each group. The association with previous independent in vitro studies is also shown.

Functional assignments of the transcriptional markers of both the ARTP and in vivo induced artemisinin responses revealed by this study support their role in key biological processes aligned with both *PfK13*-dependent and independent mechanism(s) of artemisinin resistance suggested by previous in vitro studies (summarized in Fig. 4). First, *PfK13*, the key causal factor in artemisinin resistance, was shown to localize predominantly to the cytostomes, possibly regulating endocytosis of hemoglobin^55^. *PfK13* mutations lead to a reduced rate of endocytosis and thus hemoglobin digestion, which in turn lessens the bioactivation of artemisinin as a result of lower levels of Fe^2+^ in the parasite cytoplasm. We observed baseline upregulation of factors involved in hemoglobin degradation and their suppression in sensitive parasites after 6 h in vivo treatment. Second, reductions of the *PfK13* protein levels, resulting from the mutations, were also shown to suppress the proteotoxic shock and subsequent cell death normally induced by artemisinins^56,57^. This suppression can be reversed by proteasome inhibitors in both *P. falciparum*^58^ and *P. berghei*^59^. We found marked transcriptional suppression of protein synthesis, folding, and turnover including the core subunits of the proteasome (Supplementary Data 2 and Fig. 4). Finally, PfK13 SNP-driven reduction of hemoglobin digestion and thus cytoplasmic amino acid levels can be mitigated by upregulation of nutrient permeable channels (NPC) of the parasitophorous vacuolar membrane^60^. Here we observed upregulation of at least one of the NPC encoding gene, *exp1*, that has the potential to alleviate fitness cost caused by the PfK13 mutations. In addition to transcriptional characteristics traced directly to PfK13 function, we observed transcriptional variability of factors involved in transcription, mRNA processing, ribosomal biogenesis (RiBi), translation, translational translocation, and protein transport (Supplementary Table 2 and Supplementary Data 2). This is also consistent with our earlier TPAS study of the TRACI samples where we observed upregulation of the UPR that is canonically related to proteotoxic shock response^40,41^. Third, PfK13 was also shown to function as an adaptor for the cullin3-RING-E3 ubiquitin ligase targeting a specific set of proteins for proteasome-mediated degradation^61^. Mutated forms of PfK13 fail to recognize their targets including phosphoinositide 3-kinase (PI3K), which subsequently leads to an increase in PI3P vesicles that alters the rate of hemoglobin endocytosis^62^. A set of genes involved in vesicular trafficking and the underlying lipid metabolism was also found to be transcriptionally associated with artemisinin resistance in this study (Supplementary Table 2 and Fig. 4). Fourth, *PfK13* mutations appear to mediate rewiring of the *P. falciparum* metabolic programme to higher levels of survival^50^. This involves mainly induction of mitochondrial processes including damage sensing and oxidative stress response that counteract artemisinin induced oxidative and alkylation activity^58^. In line with this, induced oxidative stress response was also detected in two independently derived in vitro *P. falciparum* artemisinin-resistant lines^51,63^. One of the chief functions of the parasite mitochondria lies in regeneration of ubiquinone, a necessary ECT component in pyrimidine biosynthesis pathways^64^ which was also shown to be altered by *PfK13* mutations^50^. Here we observed transcriptional variations of multiple REDOX and mitochondria-related factors including purine/pyrimidine synthesis pathway-related markers (OPRT, and HGPRT) suppressed in susceptible parasites but also several downregulated transcripts pertaining to ECT (SDHB, CytC) and TCA-metabolism (Supplementary Data 2). Fifth, several genes encoding proteins exported to the host cell cytoplasm were found amongst the transcriptional resistance markers. These include several members of the FIKK and PHIST gene families that are mostly implicated in host-cell remodelling affecting rigidity and/or cytoadhesion of the infected RBCs^65–68^. This could suggest that altered host-parasite interactions might contribute to artemisinin resistance; albeit indirectly by reducing parasite clearance by the spleen. Given the functional diversification of the FIKK and PHIST as well as other *P. falciparum* gene families annotated as exported proteins their direct role in artemisinin resistance mechanisms cannot be ruled out. Several of the exported proteins factors as well as many of the above-mentioned biological pathways have been recently linked with artemisinin resistance in a large-scale *P. falciparum* piggyBac-transposon mutant screen^69^. Altogether these observations support the majority of previous studies implicating a wide spectrum of biological functions discovered by in vitro experiments to play specific roles in artemisinin resistance in vivo.

Since its emergence in 2009, artemisinin resistance exerted strong selection effects on the GMS *P. falciparum* population due to a continuous drug pressure resulting from intensive high-coverage treatment programmes implemented by all countries in the region^11,43,70^. There is now a clear distinction between the *P. falciparum* population in the wGMS (Myanmar, Thai-Myanmar border, and Bangladesh) and eGMS (Cambodia Laos and Southern Vietnam), the latter of which likely provided the supporting genetic background for artemisinin resistance^40^. Moreover, substantial founder population effects were detected in eGMS also likely as a result of drug selection^71^. The main example represents the recent selective sweep of the KEL1/PLA1 parasite line throughout eGMS during 2013–2016; prior to the TRACII epidemiological survey^11,43^. Indeed, the TRACII *P. falciparum* cohort used in this study reflected both the w/eGMS segregation and KEL1/PLA1 selection sweep. This provided us with the unique opportunity to study artemisinin resistance-associated transitional changes at its origin, but also presented a major challenge due to the complex population structure. To overcome this, we implemented multiple steps during data analysis. In addition of the FPR-based differential gene expression pipeline (Fig. 1b, c), we carried out multiple confounding factor evaluations including tSNE and PCA with the whole transcriptome dataset (Supplementary Fig. 3) and subsequently UMAP, genetic/transcriptional distance ratio, and the Hamilton’s founder subpopulation with the ARTP (Fig. 2b). Notwithstanding the possible weakness of each analytical method, the combination of these provides a strong confidence that identified 156 genes can be linked to putative multifaceted artemisinin resistance mechanism. However, it is feasible to expect that there are more genes contributing to artemisinin resistance that were not uncovered by this work. This is likely due to the challenging nature of the TRACII epidemiological datasets that is characterized by a strong population structure (Fig. 1a) that forced us to develop a highly stringent bioinformatics pipeline to overcome this confounding factor. As a result, we focused on transcriptional changes of individual genes instead of gene groups capturing entire biochemical and cellular pathways that were a subject of our previous TPAS studies with TRACI cohort^40,41^. In the TRACI dataset, we identified inductions of multiple pathways related to the UPR and REDOX metabolism and suppression of multiple processes facilitating the parasite developmental progression (deceleration of the IDC). These results were reproduced in the TRACII only partially. However, multiple genes related to UPR, REDOX as well as IDC progression identified amongst the ARTP genes signal that these pathways still play a key role in artemisinin resistance. Future GWAS and TPAS using broader epidemiological cohorts will provide deeper insight into the multifaceted artemisinin resistance mechanism relevant to clinical settings.

In contrast to transcriptome-based analyses, GWAS have until now failed to identify such factors making limited observations of direct links between the molecular effectors of artemisinin resistance in vivo*;* with the exception of the *PfK13* SNP^32^. Although it is still possible that more mutations in the coding regions may contribute to artemisinin resistance they were not detected because of typical sample size limitations of GWAS^39^; in the context of a highly complex *P. falciparum* population structure in the GMS^71^. Our result strongly suggests that many causative mutations might be situated within DNA regulatory elements affecting the regulation of gene expression. It was previously shown that the majority of sequence polymorphisms in *P. falciparum* occurs in the noncoding regions accumulated particularly around core promoter regions as Insertion/Deletion within Short Tandem Repeats (STR)^72^. The high AT content (~85%) of the *P. falciparum* noncoding regions hampered most whole-genome sequencing efforts on field samples done up until now. However, the results of this study make a case for synergizing GWAS and TPAS (combined into eQTL studies) as a potential strategy to monitor the spread and evolution of malaria drug resistance in the future genomic epidemiological surveys.

## Methods

### Ethics statement

All the samples used in this study were collected with written informed consent from the patients or their legal guardians. All protocols were approved by the appropriate national ethics committees or the Oxford Tropical Research Ethics Committee.

### In vivo sample collection

All samples used in this study were collected from patients involved in Tracking Resistance to Artemisinin Collaboration 2 (TRAC2), a multi-site trial that took place between Aug 7, 2015, and Feb 8, 2018. The total number of 1100 patients with uncomplicated *Plasmodium falciparum* malaria were recruited in eight countries. In the presented study 680 patients’ samples from 6 countries and 13 sites across Southeast Asia Greater Mekong Region were analyzed (Ramu in Bangladesh; Thabeikkyin, Pyay, Pyin Oo Lwin and Ann in Myanmar; Phusing and Khun Han in Thailand; Pailin, Pursat, Ratanakiri, and Preah Vihear in Cambodia; Sekong in Laos; Bin Phuoc in Vietnam). All details regarding samples collection, site locations, inclusion criteria, parasitemia assessment, and given treatments were published previously^2^. In brief, samples were collected from venous blood of malaria-infected patients at admission to the clinic (Hour 0, 0 h) and 6 h after their respective treatment (Hour 6, 6 h). Depending on their location, patients were randomly assigned different double or triple Artemisinin Combination Therapy (ACT) as follows: Thailand, Cambodia, Vietnam, and Myanmar - dihydroartemisinin-piperaquine or dihydroartemisinin-piperaquine plus mefloquine; Cambodia - artesunate-mefloquine or dihydroartemisinin-piperaquine plus mefloquine; Laos, Myanmar, Bangladesh - artemether-lumefantrine or artemether-lumefantrine plus amodiaquine. Following collection, blood was subsequently depleted of plasma and buffy coat. Hour 0 samples were additionally purified from white blood cells (WBC) using CF11 columns using the Worldwide Antimalarial Resistance Network (WWARN) protocols. 0.2–0.5 mL of packed red blood cells (pRBC) from each sample was homogenized by mixing with 10 volumes of TRIzol (Invitrogen), frozen at −80 °C, and then shipped to Nanyang Technological University, Singapore.

### RNA isolation

All clinical samples mixed with TRIzol were supplemented with 5 volumes of chloroform (Merck) and processed as per the manufacturer’s instructions to obtain phase separation. Top (aqueous) phase was mixed 1:1 (v/v) with 100% analytical grade ethanol (Merck) and RNA was extracted using the Direct-Zol-96 extraction kit (Zymo) following the manufacturer’s guide. For this study, we have adopted the RNA extraction kit to a high-throughput EVO 200 robotic platform (Tecan). RNA integrity was assessed using Agilent Bioanalyzer (RIN) (Agilent), concentration was measured using Qubit RNA Broad Range kit (Invitrogen) and RNA contamination was estimated with Nanodrop (Thermo Fisher)^42^.

### cDNA synthesis and microarray hybridization

For each sample, 250 ng of total RNA was used for subsequent reverse transcription reaction followed by 19 rounds of PCR amplification using modified Smart-Seq2 method^73^. In brief, oligo-dT30 (IDT Asia) was used as a primer to enrich for mRNA and avoid ribosomal rRNA amplification (AAGCAGTGGTATCAACGCAGAGTACT30VN; V = A,C,G; N = A,C,G,C). The amplified product was purified using Ampure XP magnetic beads (Beckman Coulter) on Microlab Nimbus robotic platform (Hamilton) and 100 ng of cDNA was used for subsequent 10 rounds of amplification to generate aminoallyl-coupled cDNA for the hybridizations^74,75^. 17ul (~5 µg) of each Cy-5-labelled (GE Healthcare) cDNA of patient’s sample and an equal amount of Cy-3-labelled (GE Healthcare) cDNA of the reference pool were then hybridized together on our customized microarray chip using commercially available hybridization platform (Agilent) for 20 h at 70 °C with rotation at 10 rpm. Microarrays were washed and immediately scanned using Power Scanner (Tecan) at 10 µM resolution and with automated photomultiplier tubes gain adjustments to balance the signal intensities between both channels. The reference pool used for microarray was a mixture of 3D7 parasite strain RNA collected every 6 h during 48 h of the full intraerythrocytic developmental cycle.

### RNA-sequencing

Purified 1 ng of cDNA was used to generate sequencing libraries using Illumina Nextera XT kit as described in manufacturer’s protocol. Purified cDNA libraries were analyzed on the Bioanalyzer High-Sensitivity DNA chips (Agilent), subsequently pooled (20–24 samples per lane) and sequenced on Illumina HiSeq4000 platform generating 150 bp paired end reads with 110 Gb data output generated per lane.

### In vitro culture

Plasmodium falciparum 3D7 strain was obtained from BEI Resources (MRA-102) and maintained in purified human pRBC in RPMI 1640 medium (Gibco) supplemented with Albumax I (Gibco) (0.25%), hypoxanthine (Sigma) (0.1 mM), Sodium bicarbonate (Sigma) (2 g/L), and gentamicin (Gibco) (50 μg/L). Cultures were kept at 37 °C with 5% CO_2_, 3% O_2_, and 92% N_2_. Culture media were replenished every 24 h. Freshly washed pRBC were added to the culture when necessary. Both parasitemia and parasite morphology were assessed by microscopic examination of blood smears stained with Giemsa (Sigma).

### Intraerythrocytic asexual developmental cycle reference time course (IDC)

IDC reference time course data were obtained from the study published previously^42^. In brief, prior to time-point experiment, parasites were double-synchronized with 5% sorbitol solution to achieve a synchrony of +/−6 h and cultured under constant agitation. For a sampling of highly synchronous parasites during the asexual life cycle, the first time point was considered as the TP1 (Time Point 1) when > 95% of early ring stage parasites (approx. 4 hpi) were present in the culture. Starting from TP1, parasites were collected every 2 h for 25 successive time points. The total of 24 time points were used here to build an asexual reference transcriptome generated using two different platforms: microarrays and RNA-seq.

### Raw transcriptome of *P.falciparum* parasites

The transcriptome was generated by quantifying the total RNAs for each parasite sample using two-colour microarray or RNA-seq or both. Additionally, control samples derived from ring stages 3D7 lab strain were quantified together with those clinical samples in microarray method. It was made up of a total of 30 samples for pre-treatment and 27 samples for post-treatment.

The raw data of microarray was acquired using GenePix Pro v6.0 software (Axon Instruments). Then the signal intensities were Loess-normalized within arrays followed by quantile-normalization between samples/arrays using Limma package of R^76^. Missing values were assigned to weak signal probes showing median foreground intensity less than 1.5-fold of the median background intensity at either Cy5 (sample RNA) or Cy3 (reference pool RNA) channel. Each gene expression was estimated as the average of log2 ratios (Cy5/Cy3) for probes representing it.

For RNA-seq, raw reads were trimmed to remove sequencing adapters, amplification primers, and low-quality bases from 3′ ends. HISAT2 aligner^77^ was used to perform alignment to the *P.falciparum* genome downloaded from geneDB database in version of March 2018. BEDTools^78^ was applied to calculate the read counts for each annotated transcript based on only uniquely mapped reads with pairs in proper orientation. At last, the transcriptional level was estimated for each gene by calculating the Fragments per kilo base per million mapped reads (FPKM) at the gene.

### Developmental stage estimation

For each parasite sample, we estimated the asexual age (hours post-invasion, hpi) and the proportion of gametocytes using the method described in Zhu et al’s and Lemieux et al’s works^40,79^. In brief, the expression value of a gene, *E*_*g*_, is assumed as a sum of the expression in asexual stage of hpi h, denoted as *x*_*g*_(h), and the average expression of sexual stages (from the 5th to 12th day during gametocytes developing), denoted as *z*_*g*_, mixed in certain proportions. The mixture model is presented in formula as:

1 $${E}_{g}=(1-\alpha ) * {x}_{g}{{{{{\rm{(h)}}}}}}+\alpha * {z}_{g}+{\varepsilon }_{g}$$

where α is the proportion of gametocytes to the total parasite count (sum of sexual and asexual parasites) and the *ε*_*g*_ is the associated error term. *x*_*g*_(h) is estimated by the reference transcriptome generated during asexual intraerythrocytic cycle in *P. falciparum* 3D7 strain which has a total of 25 time points with 2 h intervals of 48 h, accessible via GEO accession number GSE149865. To obtain a higher resolution of the asexual reference transcriptome for stages estimation, 24 time points of the data (the 9th time point removed due to its big dissimilarity to others) were interpolated into 240 data points by smooth splines method. *z*_*g*_ is estimated by the identical reference gametocyte transcriptome described in Zhu et al’s work^9^, accessible via GEO accession number GSE121505. In practice, samples were normalized by control samples across batches by adding a scaling factor for each batch before applying the prediction model. The scaling factor was estimated for each batch by calculating the average expression difference between controls and the reference at corresponding ages. Finally, we evaluated the log-likelihood values over a grid of mixtures for varying the gametocyte proportion *α*, and hpi h, for each sample. The results of estimated hpi and gametocytes proportion were listed in Supplementary Data 1.

### Transcriptome filtering

To achieve high quality data for the following analyses, we pruned the sample set according to their transcriptome heterogeneity and discarded samples with low signal intensities. In practice, outlier samples were removed if they were distinct from the majorities with < 10 cohort samples at the similarity threshold for grouping. The threshold was determined by the average similarity of controls to clinical samples using Spearman’s *rho*. Second, we removed samples displaying extremely low intensities (mode of the intensity < 10) of Cy5 signal on the microarray (Supplementary Fig. 8). In addition, to reduce the prediction errors (if any) affecting on the following study, we removed 2 samples having very high PC½ as 13.1 and 19.3 h (>2 times Median Absolute Deviation to the median), and 24 samples with extremely high gametocytes prediction (>18%, 3times MAD to the median) as most of the parasite samples exhibiting a low even zero proportion of gametocytes. We found all the discarded samples at this end mostly having a low parasitemia or high human content. Finally, with the microarray method, we identified a transcriptome of 4779 genes presented across > 75% of the 577 samples pre-treatment and 4714 genes across 459 samples post-treatment. Among the total of 577 pre-treatment samples and 459 post-treatment samples, 438 samples were paired (collected from the identical patient). With the RNA-seq method, we asked for at least 1 M uniquely mapped reads in a library to call for the transcriptome. Overall, we identified the transcriptome of 4305 genes with > 75% representation for 188 samples pre-treatment and 3923 genes for 159 samples post-treatment for further analyses. The data is accessible in Gene Expression Omnibus (GEO) database via the series accession number GSE149735 for microarray and GSE169520 for RNA-seq.

### PCA and population transcriptomic analysis

Principle Component Analysis (PCA) was applied to the ^(bl)^0 h transcriptome data set. We inspected the top 12 PCs for the following association analysis because each of the rest PCs contributed to < 1% of the overall transcriptome variations. The top 12 PCs were tested against all the clinical and technical factors collected during sample processing to assess the potential environmental influence on the population transcriptomic structure. For the factors represented in categorical variables like sampling sites, parasite lineages, and patient’s gender, ANOVA test was used for assessing the statistical significance of associations. For other factors in continues/discrete variables like parasitemia, hpi and patient enrolment time etc., linear regression was used to test the statistical relationship between each factor and each PC with Spearman’s *rho* calculated for each pair of variables as well. The results are visualized in Supplementary Fig. 3a.

The regression analysis revealed that parasite age (hpi) significantly (*p* = 7.96e−283) correlated to the top PC (PC1) with showing a high Spearman’s *rho* as 0.87 in the ^(bl)^0 hr data set by microarray and 0.85 by RNA-seq. PCA of all samples including both ^(bl)^0 h and ^(tr)^6 h resulted in similar high correlations with displaying *rho* as 0.78 in microarray and 0.76 in RNA-seq (Supplementary Fig. 3b). It indicates that hpi is the major factor distinguishing parasites transcriptome in field. Correlation was also observed between the PC2 and the ratio of parasite to human content; the PC6 and estimated gametocytes proportion but the Spearman’s *rho* was dropped to 0.53 and 0.52, respectively. These two factors (parasite/human ratio and gametocytes fraction) can interact with other experimental or environmental conditions to drive the minor (compare to hpi) differences in parasites transcriptome. For this study, we considered only hpi as the major factor contributing to gene expression variation across nature parasites and used it as a major predictor variable in modelling gene expression for the further regression analysis. The potential for expression variation caused by other factors, like sampling site and parasite lineage, are analyzed for particular genes associated to resistance in the following studies.

To visualize the parasite population structure in a two-dimensional map, t-distributed stochastic neighbour embedding was applied to the PC2-12 with PC1 excluded to minimize the hpi effects. It was implemented in R with the M3C package^80^.

### The PC space projection

We performed PCA to the reference transcriptome of 3D7 parasite stain at ring stages together with the average transcriptome of 3D7 mature gametocytes (5th–12th day during development). The resulted PC1 clearly distinguishes the sexual and asexual stages, as well as the PC2, reflects the ring-stage parasite development as it obviously separates the 8 ring-stage transcriptomes with 2 h difference in between. With the space of PC1 vs PC2, parasite age can be visualized without bias by transcriptome projection. Therefore, we normalized all the clinical samples to the centre transcriptome derived from the above PCA and rotated it according to the PC1 and PC2 loadings. At last, the 577 pre-treatment samples and 459 post-treatment samples were projected onto the PC space showing an obvious age window shift in the parasites after drug treatment (Fig. 3a).

### Population stratification

The SNP information was provided by the Wellcome Sanger Institute. We extracted out a high-quality set of 7009 SNPs for the population stratification analysis here. The filtering criteria are as follow: (1) each polymorphism was covered by at least 10 reads; (2) the genotyping quality score was greater than 30 by GATK; (3) the minor allele count was at least 5 across the genotyped samples we studied, and the minor allele frequency was at least 0.05. (4) Only SNPs showing two alleles with our studied samples are included; (5) only SNPs in coding regions were included; (6) samples with more than 50% high quality SNPs missing were removed from this analysis. At last, 7009 SNPs and 528 isolate samples were used to determine the population stratification using Plink multidimensional scaling function with IBS pairwise distances. UMAP algorithm was applied to visualize the result in Fig. 2b. The UMAP-2 majorly distinguished the eGMS parasites from the wGMS and the UMAP-1 reflected the genetic difference within each region’s parasites.

To measure the genetic differences between the ARTP clusters (Fig. 2b), we constructed the genetic distance matrix using the IBS pairwise distances. And the pair-wise distance was calculated between individuals within each cluster and between clusters. The ratio of the average distance between within-cluster, *D(within-cluster)*, and between-cluster, *D(between-cluster)* individuals was used to indicate genetic differences between clusters and compare to the ratio calculated from transcriptional distance matrix.

### Transcriptome-phenotype association study

Transcriptome-phenotype association study (TPAS) was carried out in order to call for the marker candidates whose mRNA levels were positively or negatively associated with artemisinin resistance. Since parasite age contributed the most to expression variation across clinical samples, we designed a generalized additive model with the age/hpi specified in a loess function to test the expression-resistance association over the dynamic relationship between expression and age. The regression analysis formula for each gene is represented as:

2 $$E={\beta }_{0}+{\beta }_{1}* f{{{{{\rm{(hpi)}}}}}}+{\beta }_{2}* hf+\varepsilon$$

Where *E* denotes gene expression across samples, *f(*hpi*)* denotes the function of hpi which is loess regression here, *hf* denotes the variable of PC½, *β*_0,1,2_ represents the parameters of intercept and slopes to predict and *ε* is the error terms. Therefore, the resulted *p*-value of this regression analysis reflects the relationship between expression residuals of age fitting to the half-lives (PC½), alternatively the expression-resistance association independent of age.

With this approach, we tested the expression-resistance association for all the genes, 4779 genes with microarray and 4714 genes with RNA-seq, individually. To correct for the multiple testing, we estimated FDR for each gene by 1000 times permutation constructing a null *p* distribution based on testing the association of expression to randomized PC½ values (Fig. 1c). In addition, to address the cofounding relation between artemisinin resistance (PC½ > 5 h) and parasite genetic lineage which also largely coincided with the geographical region (w/eGMS), we estimated a FPR value for each gene to control the type I error by 100 times permutations. In each permutation, the lineage structure was maintained and PC½ values were randomized amongst the parasites within each lineage. The resulted null *p*-values reflect the significance of expression-lineage associations independent of PC½ and the FPR calculated from the null p distribution (Fig. 1b) reflect the probability of expression-resistance association caused by expression-lineage relationship. At last, FPR < 0.05 (95% confidence) was applied to define the robust expression-resistance associations beyond parasite lineage effect. We plotted the expression residuals against the original and randomized PC½ values for PHISTa gene (shown in Fig. 1b) to illustrate the randomization procedure, also for three example genes with significant expression-resistance associations (FDR < 0.05) and different levels of FPR (0.01, 0.53, and 1) together with other two genes with FDR > 0.5 and FPR < 0.01 for reference (Supplementary Fig. 5).

We applied this approach to microarray and RNA-seq data separately. The results agreed to each other with showing a high correlation (Pearson correlation coefficient = 0.68) of the average expression fold change of resistant/susceptible between the two datasets (Supplementary Fig. 6). We merged the markers defined by microarray or RNA-seq excluding 10 genes that displayed conflicting directions of expression changes in resistant parasite in two techniques. Finally, our approach determined 69 genes upregulated and 87 genes downregulated in the resistant parasites at FDR < 0.05 (corresponding *p* < 1e−10 in microarray, *p* < 1e−6 in RNA-seq) and FPR < 0.05.

We applied the same TPAS pipeline to the TRACI data and reanalyzed 824 samples collected during 2011–1013. It resulted in 61 expression upregulation and 63 downregulation associated to artemisinin resistance with the identical criteria above (FDR < 0.05 & FPR < 0.05, corresponding *p* < 8.5e−6). This result significantly overlaps that from TRACII with 14 upregulation and 12 downregulation in common (binomial test *p* < 1e−9).

### ROC curve

ROC curve was applied to access the ability of distinguishing the resistance and susceptible parasites for individual expression markers, combination of markers, and the C580Y mutation. For each gene, the sensitivity and specificity of classifying PC½ into groups of > 5 h or < 5 h was calculated at a varied expression threshold which has 20 data points evenly distributed from the minimum to the maximum expression value observed in the studied parasites. For the combination of markers, we use the measurement as the sum up expression values of the 69 upregulations with a subtraction of the sum up of the 87 downregulations. Then the varied threshold of 20 points was set similarly as the above. Since the combination of multiple genes can always perform better than single, we also generate ROC curve for 10 randomly selected gene sets. Each of the 10 gene sets combined 69 genes randomly selected from genes over-expressed in resistance parasites and 87 random under-expressed genes. The result suggests the combination of the 156 markers having the best performance comparing to each single marker or random genes and it is the most close to the performance of the C580Y mutation (sensitivity = 0.75 and specificity = 0.91, Fig. 2a).

### ARTP clustering

To investigate the resistance-associated transcriptome structure, we first defined the artemisinin resistance-associated transcriptional profiles (ARTP) for each parasite sample using the 156 marker genes. To obtain expression levels with hpi effects maximumly reduced, we second extracted out the expression residuals from the formula (2) with the hpi function fitting only for each gene. The expression residuals were normalized for the 323 resistant parasite samples (PC½ > 5 h and from eGMS) against that of 104 susceptible samples (PC½ < 5 h and from wGMS) by calculating z-scores to represent the number of standard deviations by which the expression of the studied gene in resistant parasite was above or below the mean in susceptible parasites. Next, Euclidean distance was applied to the similarity matrix of ARTP to construct the sample distance matrix and the Ward’s method was used to obtain the dendrogram of clustering tree for the 323 resistant parasite samples (Fig. 2b). The six clusters shown in Fig. 2b were defined by the tree cutting at the 1/6 of tree height using the “cutree” function in R stats package.

To measure the transcriptional differences between clusters, we used the Pearson distance of the ARTP to construct the transcriptional distance matrix. And the pair-wise distance was calculated between individuals within each cluster and between clusters. The ratio of the average distance between within-cluster, *D(within-cluster)*, and between-cluster, *D(between-cluster)* individuals was used to indicate transcriptional differences between clusters and compare to the ratio calculated from a genetic distance matrix.

### In vivo transcriptional response measurement

The transcriptional response to artemisinin was inspected for the field *P.falciparum* parasites by comparing the ^(tr)^6 h sample set to the ^(bl)^0 h sample set. Grouping the ^(bl)^0 h samples by lineage and resistance status level (PC½ greater/smaller than 5 h) revealed two largest groups which are KEL1PLA1 parasites with PC½ > 5 h (37% of the total 577 samples) and *PfK13* WT parasites with PC½ < 5 h (29% of 577). Excluding the lineage unknown samples, all the rest groups contain < 6% of the total samples. For this study, we performed the comparative transcriptional analysis specifically for the *PfK13* WT samples with PC½ < 5 h (the susceptible parasites) and the KEL1PLA1 samples with PC½ > 5 h (the resistant parasites).

We adjusted the above regression model of formula (2) to better present the data of ^(tr)^6 h and ^(bl)^0 h samples as:

3 $$E={\beta }_{0}+{\beta }_{1}* f{{{{{\rm{(hpi)}}}}}}+{\beta }_{2}* {{{{{\rm{treatment}}}}}}+{\beta }_{3}* {{{{{\rm{resistance}}}}}}\_{{{{{\rm{status}}}}}}+\varepsilon$$

Where *treatment* indicates patient treatment condition which is pre-treatment (^(bl)^0 h) or post-treatment (^(tr)^6 h), and *resistance_status* indicates PC½ greater/smaller than 5 h. To discover the distinct transcriptional response for resistant and susceptible parasites individually, we performed a comparative analysis for each parasite group (resistant/susceptible). We aimed to define top drug-response genes for each parasite group independent of age and batch effects (due to the collection and transportation issue with the ^(bl)^0 h and ^(tr)^6 h sample set, the treatment condition here unavoidably confounded with the batch of transcriptome measurement). To achieve that, we first extracted the expression residuals from the model (3) with the loess fit only which maximumly removed the expression variations caused by age from the raw data. Second, we conducted Mann–Whitney test on the expression residuals between treatment conditions to compare 216 ^(bl)^0 h to 180 ^(tr)^6 h samples for resistant parasites and 168 ^(bl)^0 h to 130 ^(tr)^6 h samples for susceptible parasites with microarray measurement. To balance the sample size differences, subsampling was applied to 130 samples 100 times per gene to obtain the *p-*value at 80% confident level. For the multiple test correction, we calculated FDR for each gene using the distribution of 500,000 null *p*-values generated from expression permutation based on all ^bl)^0 h and ^(tr)^6 h samples. To control the effect of treatment/batch, the structure of the treatment condition was maintained during each time permutation.

We repeated the same analysis with the RNA-seq data. Mann–Whitney test was conducted to compare 67 ^(bl)^0 h to 55 ^(tr)^6 h samples for resistant parasites and 57 ^(bl)^0 h to 46 ^(tr)^6 h samples for susceptible parasites. And the *p-*values were obtained by 100 subsampling of 46 samples per gene. The result was merged with that from microarray for susceptible and resistance group, respectively, at FDR < 0.05.

By this approach, we identified 20 significantly induced genes and 73 repressed genes upon drug in the KEL1PLA1 resistant parasites and 33 induced genes and 106 repressed genes in the WT susceptible parasites (FDR < 0.05, corresponding *p* < 1e−14). Significant common response genes were observed between the resistant and susceptible parasite samples which included up to 12 induced and 34 repressed genes (Fig. 3b and Supplementary Data 2).

### Statistics and reproducibility

We analyzed 577 samples pre-treatment with 459 samples post-treatment using microarray method and 188 samples pre-treatment with 159 samples post-treatment using RNA-seq method. Due to the nature of this study as a part of the larger clinical trial, no biological replicates were used. In this study, binomial test or hypergeometric test was applied to the enrichment analyses; ANOVA test, Pearson Correlation Coefficient, and linear regression were performed for association or correlation testing between factors. We used FDR and FPR to help control for false positives that resulted in 156 resistance-associated marker genes defined at FDR < 0.05, FPR < 0.05, and the corresponding *p* < 8.5e−6. For the differential expression analysis, Mann–Whitney test was applied for group comparison. Differentially expressed genes were defined at FDR < 0.05 and the corresponding *p* < 1e−14. Subsampling method was used to balance sample size difference and the *p*-values were obtained at 80% confidence level. All details regarding the statistics of this study can be found above in the description of each respective analysis.

### Reporting summary

Further information on research design is available in the Nature Research Reporting Summary linked to this article.

## Supplementary information

Supplementary Information

Editorial Assessment Report

List of Supplementary Information

Supplementary Data 1

Supplementary Data 2

Reporting Summary

## Data Availability

All data is available in the main text or the supplementary materials or from the corresponding author upon request. All transcriptome data used in this study are available at NCBI’s Gene Expression Omnibus (GEO) database with the series accession number GSE149735 for microarray and GSE169520 for RNA-seq.
