# Supplementary material for: Artemisinin resistance in the malaria parasite, Plasmodium falciparum, originates from its initial transcriptional response
Source: Commun Biol. 2022 Mar 28;5:274. doi: 10.1038/s42003-022-03215-0 (PMC8960834; doi:10.1038/s42003-022-03215-0)
Supplement: Supplementary file 1 — Supplementary Information [file 42003_2022_3215_MOESM1_ESM.pdf]

## Supplementary information

Supplementary Table 1: Summary of samples in this study.

Supplementary Table 2: Top selected transcriptional resistance markers derived from this study.

Supplementary Figure 1: Transcriptional profiles of the studied *P.falciparum* parasites.

Supplementary Figure 2: Distribution of the estimated age, gametocytes fraction and parasite clearance half-life.

Supplementary Figure 3: PCA of the parasite transcriptome.

Supplementary Figure 4: Expression level correlates to age/hpi for most of the *P. falciparum* genes.

Supplementary Figure 5: Examples for FPR calculation.

Supplementary Figure 6: Consistent results of transcriptome analysis across platforms

Supplementary Figure 7: Compare pairwise and non-pairwise analysis of DE genes for drug response.

Supplementary Figure 8: Defined intensity threshold for microarray-generated transcriptome filtering.

Supplementary Data 1: Parasite age and gametocyte proportion estimation

Supplementary Data 2: Expression markers of resistance and drug response genes

## Supplementary Tables

Table S1

| | | $^{(bl)}$ *0hr* | | | $^{(tr)}$ *6hr* | | |
|---|---|---|---|---|---|---|---|
| | | Total | Microarray | RNA-seq | Total | Microarray | RNA-seq |
| | | 584 | 577 | 188 | 461 | 459 | 159 |
| Bangladesh | 1.Ramu | 115 | 110 | 47 | 91 | 91 | 43 |
| Myanmar | 2.Thabeikkyin | 22 | 21 | 7 | 15 | 15 | 7 |
| | 3.Pyin Oo Lwin | 12 | 12 | 3 | 11 | 11 | 2 |
| | 4.Pyay | 27 | 27 | 12 | 24 | 24 | 9 |
| | 5.Ann | 26 | 26 | 2 | 19 | 19 | 2 |
| Laos | 6.Sekong | 10 | 10 | 1 | 0 | 0 | 0 |
| Thai-Cambodia border | 7.Pusing | 31 | 31 | 12 | 30 | 30 | 13 |
| | 8.Khun Han | 6 | 6 | 0 | 3 | 3 | 0 |
| Cambodia | 9.Preah Vihear | 7 | 7 | 2 | 5 | 5 | 2 |
| | 10.Ratanakiri | 80 | 80 | 20 | 57 | 57 | 12 |
| | 11.Pailin | 57 | 57 | 32 | 51 | 51 | 30 |
| | 12.Pursat | 78 | 78 | 20 | 64 | 63 | 14 |
| Southern Vietnam | 13.Binh Phuoc | 113 | 112 | 30 | 91 | 90 | 25 |

**Supplementary Table 1:** Summary of samples in this study.

| Gene ID | Description | Symbol | TPAS at baseline (with p-values) | *In vivo* response (with p-values) | | Link with other studies | Expression across 3D7 IDC^ |
|---|---|---|---|---|---|---|---|
| | | | | *S* | *R* | | rg trp sch rg |
| **Ribosomal structure and function** | | | | | | | |
| PF3D7_1309100 * | 60S ribosomal protein L24 | RPL24 | ↓ 1.33E-11 | ↓ 1.46E-11 | ↓ 3.89E-01 | 1↓, 5 | |
| PF3D7_1142500 | 60S ribosomal protein L28 | RPL28 | ↓ 8.74E-15 | ↓ 2.41E-08 | - | 1↓, 5 | |
| PF3D7_0722600 | U3 small nucleolar RNA-associated protein 7 | UTP7 | ↑ 2.10E-21 | ↓ 1.59E-01 | ↓ 5.53E-05 | 5 | |
| PF3D7_0106400 | pre-rRNA-processing protein TSR2 | TSR2 | ↓ 3.49E-25 | ↓ 7.24E-18 | ↓ 2.79E-03 | 1↓, 4 | |
| **Protein synthesis** | | | | | | | |
| PF3D7_1338300 * | elongation factor 1-gamma | EF-1gamma | ↓ 1.82E-14 | ↓ 1.53E-15 | ↓ 6.52E-11 | 1↓, 5 | |
| PF3D7_1464600 | serine/threonine protein phosphatase UIS2 | UIS2 | ↑ 6.33E-12 | - | ↑ 1.01E-01 | 4, 5 | |
| **Mitochondrial processes / redox** | | | | | | | |
| PF3D7_0802000 * | glutamate dehydrogenase | GDH3 | ↑ 1.64E-10 | ↓ 1.52E-01 | ↓ 8.88E-13 | 1↑, 5 | |
| PF3D7_0926700 | glutamine-dependent NAD(+) synthetase | NADSYN | ↓ 8.38E-15 | ↓ 6.47E-04 | ↑ 1.91E-02 | 1↓, 4 | |
| PF3D7_1404100 | cytochrome c | | ↓ 2.15E-12 | ↓ 6.47E-17 | ↓ 8.63E-08 | 1↓, 2↓ | |
| PF3D7_0814900 | superoxide dismutase [Fe] | FeSOD | ↓ 7.10E-22 | ↓ 2.67E-16 | ↓ 7.10E-21 | 1↓ | |
| PF3D7_0823700 | mitochondrial import receptor subunit TOM7 | TOM7 | ↑ 3.92E-22 | ↓ 2.73E-11 | ↓ 2.15E-35 | | |
| **Protein turnover / Proteosome** | | | | | | | |
| PF3D7_0317000 | proteasome subunit alpha type-3 | | ↓ 2.66E-12 | ↓ 5.16E-17 | ↓ 5.28E-09 | | |
| **Hemoglobin degradation** | | | | | | | |
| PF3D7_1115400 | cysteine proteinase falcipain 3 | FP3 | ↑ 7.59E-09 | ↓ 6.19E-08 | ↓ 2.39E-19 | 4, 5 | |
| PF3D7_0932300 | M18 aspartyl aminopeptidase | M18AAP | ↓ 1.27E-14 | ↓ 4.93E-15 | ↓ 1.15E-16 | 1↓, 3↓, 5 | |
| **Protein folding / Chaperones** | | | | | | | |
| PF3D7_0629200 * | DnaJ protein | DnaJ | ↑ 2.24E-13 | - | ↓ 1.25E-07 | 1↑, 4, 5 | |
| PF3D7_0314000 | HSP20-like chaperone | | ↓ 5.85E-08 | - | - | 1↑, 5 | |
| PF3D7_0113700 | heat shock protein 40 type II | HSP40 | ↑ 6.33E-22 | ↑ 8.34E-05 | ↓ 1.36E-04 | 1↑, 2↑, 4 | |
| PF3D7_1372200 | histidine-rich protein III | HRPIII | ↓ 6.15E-21 | ↓ 2.73E-04 | ↓ 8.28E-01 | 1↓, 4 | |
| PF3D7_1357800 * | T-complex protein 1 subunit delta | CCT4 | ↑ 1.56E-10 | ↓ 1.53E-04 | ↓ 5.76E-11 | 1↓, 4 | |
| PF3D7_0831700 | heat shock protein 70 | HSP70x | ↓ 6.57E-12 | ↓ 1.62E-06 | ↓ 1.23E-03 | 1↓ | |
| **PV / Exported proteins** | | | | | | | |
| PF3D7_0202000 * | knob-associated histidine-rich protein | KAHRP | ↑ 1.40E-13 | ↑ 4.74E-01 | ↓ 4.48E-13 | 1↑, 3↑, 4, 5 | |
| PF3D7_0201500 | Plasmodium exported protein (hyp9) | HYP9 | ↑ 2.62E-14 | - | ↓ 2.18E-11 | 1↑, 2↑, 4, 5 | |
| PF3D7_0830900 | Plasmodium exported protein | | ↑ 4.80E-08 | ↓ 8.52E-03 | ↓ 2.92E-13 | 1↑, 4, 5 | |
| PF3D7_0102600 | serine/threonine protein kinase FIKK family | FIKK1 | ↑ 2.41E-17 | - | ↓ 3.23E-19 | 1↑, 4, 5 | |
| PF3D7_0424900 | Plasmodium exported protein (PHISTa) | PHISTa | ↑ 2.30E-09 | ↑ 1.35E-07 | ↑ 1.67E-08 | 4, 5 | |
| PF3D7_0201900 | erythrocyte membrane protein 3 | EMP3 | ↑ 1.94E-24 | - | ↓ 4.92E-11 | 1↑, 2↑, 3↑, 5 | |
| PF3D7_1001500 | early transcribed membrane protein 10.1 | ETRAMP10 | ↓ 1.51E-12 | - | - | 1↓, 2↓, 3↓, 5 | |
| PF3D7_1372000 | Plasmodium exported protein (PHISTa) | PHISTa | ↑ 7.06E-27 | ↑ 9.79E-24 | ↑ 1.76E-09 | 1↑, 2↑, 3↑, 4 | |
| PF3D7_0424700 | serine/threonine protein kinase FIKK family | FIKK4.2 | ↑ 5.17E-24 | ↑ 1.55E-11 | - | 1↑, 3↑, 4 | |
| PF3D7_1001400 | exported lipase 1 | XL1 | ↑ 1.84E-14 | ↑ 5.03E-03 | - | 1↑, 2↑, 4 | |
| PF3D7_0410000 | erythrocyte vesicle protein 1 | EVP1 | ↑ 1.32E-12 | ↑ 7.01E-11 | - | 1↑, 4 | |
| PF3D7_1301400 | Plasmodium exported protein (hyp12) | HYP12 | ↑ 1.96E-14 | ↑ 3.70E-05 | ↓ 2.47E-06 | 1↑, 4 | |
| PF3D7_0220600 | Plasmodium exported protein (hyp9) | HYP9 | ↑ 4.13E-17 | - | ↓ 7.08E-16 | 1↑, 4 | |
| PF3D7_0501200 * | parasite-infected erythrocyte surface protein | PIESP2 | ↑ 4.28E-11 | - | ↓ 8.08E-08 | 1↑ | |
| **Pyridoxine/Polyamine synthesis** | | | | | | | |
| PF3D7_0608800 * # | ornithine aminotransferase | OAT | ↑ 2.66E-12 | - | ↓ 4.19E-12 | 1↑, 5 | |
| PF3D7_0616000 | pyridoxal kinase | PDXK | ↑ 2.39E-12 | - | ↓ 4.10E-06 | 1↑, 4 | |
| PF3D7_1129000 * | spermidine synthase | SpdSyn | ↑ 5.09E-13 | - | ↓ 8.22E-07 | 4 | |
| **Trafficking** | | | | | | | |
| PF3D7_0816700 | trafficking protein particle complex subunit 2-like | TRAPPC2L | ↓ 2.81E-19 | ↓ 1.38E-23 | ↓ 7.48E-05 | 4, 5 | |
| PF3D7_1405200 | trafficking protein particle complex subunit 1 | TRAPPC1 | ↓ 1.01E-15 | ↓ 6.14E-10 | ↑ 2.52E-03 | 4 | |
| PF3D7_1442000 | ADP-ribosylation factor | | ↓ 1.24E-28 | ↓ 5.63E-16 | ↓ 3.25E-11 | 4 | |
| **Glycolysis** | | | | | | | |
| PF3D7_0626800 * | pyruvate kinase | PyrK | ↓ 1.53E-16 | ↓ 2.81E-10 | ↓ 5.25E-11 | 1↓, 5 | |
| PF3D7_0624000 * # | hexokinase | HK | ↑ 4.00E-19 | ↓ 2.46E-17 | ↓ 2.81E-16 | | |
| **Transcription** | | | | | | | |
| PF3D7_1317200 | AP2 domain transcription factor AP2-G3 | ApiAP2 | ↑ 7.12E-18 | ↑ 4.50E-07 | - | 1↑, 2↑ | |
| PF3D7_1107800 | AP2 domain transcription factor | ApiAP2 | ↑ 3.10E-07 | ↓ 1.28E-06 | ↓ 1.18E-13 | 1↑, 2↑ | |
| **Ungrouped** | | | | | | | |
| PF3D7_0819600 | conserved Plasmodium protein | | ↓ 4.56E-12 | - | - | 2↓, 3↓, 4, 5 | |
| PF3D7_1427900 | leucine-rich repeat protein | | ↓ 3.70E-11 | - | - | 1↓, 3↓, 4, 5 | |
| PF3D7_0624200 | conserved Plasmodium protein | | ↑ 1.07E-07 | ↑ 1.33E-11 | - | 1↑,4, 5 | |
| PF3D7_1454700 | 6-phosphogluconate dehydrogenase decarboxylating | 6PGD | ↓ 6.12E-13 | - | ↑ 9.28E-05 | 1↓, 4, 5 | |
| PF3D7_0814200 | DNA/RNA-binding protein Alba 1 | ALBA1 | ↓ 6.51E-17 | - | ↑ 1.52E-01 | 1↓, 4, 5 | |
| PF3D7_1473700 | nucleoporin NUP116/NSP116 | NUP116 | ↑ 6.69E-13 | ↑ 1.57E-10 | ↑ 4.35E-07 | 1↑, 2↑, 4 | |
| PF3D7_0415300 | cdc2-related protein kinase 3 | CRK3 | ↓ 4.11E-08 | - | - | 1↓, 4 | |
| PF3D7_1237700 * | conserved protein | | ↓ 1.11E-08 | - | - | 1↓, 4 | |
| PF3D7_0810500 | protein phosphatase PPM7 | PPM7 | ↓ 3.45E-12 | ↑ 6.48E-03 | ↑ 5.26E-10 | 4, 5 | |
| PF3D7_0619900 | splicing factor 3A subunit 2 | SF3A2 | ↓ 9.77E-16 | ↓ 9.61E-01 | ↓ 8.51E-04 | 4, 5 | |
| PF3D7_1468800 | splicing factor U2AF large subunit | U2AF2 | ↓ 1.04E-13 | ↓ 1.56E-06 | - | 4, 5 | |
| PF3D7_0514800 | inositol polyphosphate multikinase | | ↓ 5.79E-14 | - | ↑ 6.17E-04 | 3↓, 5 | |
| PF3D7_0404500 | 6-cysteine protein | P52 | ↑ 4.76E-13 | ↓ 1.65E-35 | ↓ 2.66E-40 | | |
| PF3D7_0211700 | tyrosine kinase-like protein | TKL1 | ↑ 1.67E-14 | ↓ 2.05E-31 | ↓ 3.21E-34 | | |

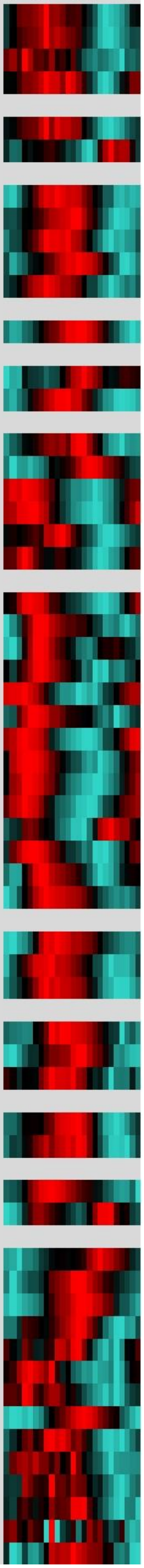

**Supplementary Table 2.** Top selected transcriptional resistance markers derived from this study. Candidates are derived from TPAS at baseline level ([bl]0hr) and from *in vivo* treatment ([tr]6hr) sample sets. Markers are grouped according to their literature-based functional assignments and cross-referenced to other independent transcriptomics studies. Putative direct artemisinin targets are indicated (*/#). For better visualization of co-expressed transcripts relative expression levels across 3D7 IDC in vitro are shown in the last column (red – upregulation, turquoise – downregulation). *Artemisinin targets by Wang, J. et al. (2015). #Artemisinin targets by Ismail, HM et al. (2016). 1) 1h DHA-treated vs. non-treated K1 at ring stage, Shaw, JP. et al. (2015) 2) 3h Artesunate-treated vs. non-treated FCR3, Natalang, O. et al. (2008) 3) Differentially expressed genes from TWAS analysis of clinical samples during TRAC1 study, Mok, S. et al. (2015) 4) Differentially expressed genes between K13 mutant and wild type strains Mok, S. et al. (2021) 5) Differentially expressed genes in in vitro Art-resistance selected 3D7 strains, Rocamora, J. et al. (2018). ^RNA-Seq IDC relative expression values from Kucharski, M. et al. (2020). R - resistant, S - sensitive, ↑ - upregulation, ↓ - downregulation, rg - rings, trp - trophozoites, sch - schizonts.

**Supplementary Figures**

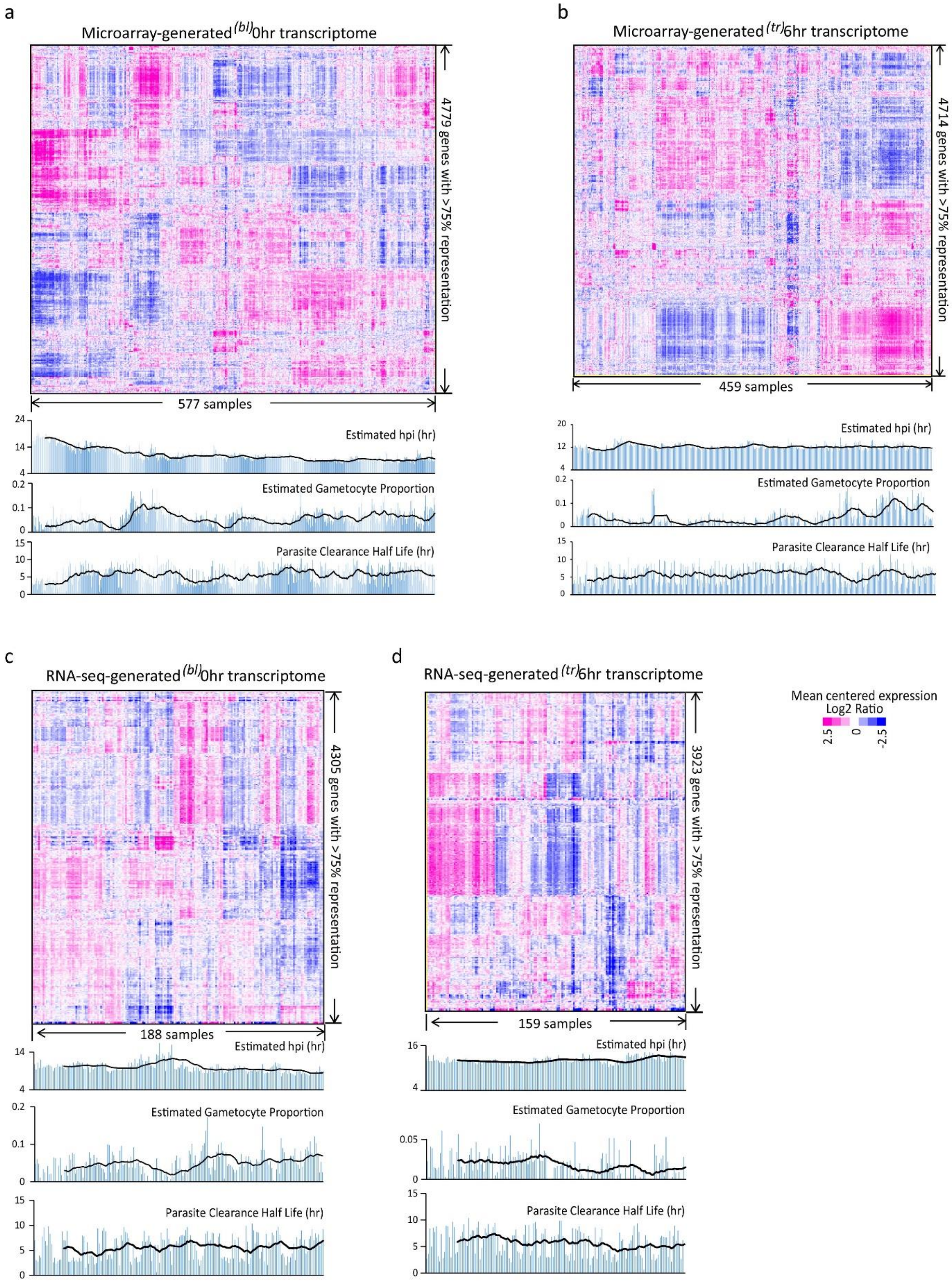

**Supplementary Figure 1**: Transcriptional profiles of the studied *P.falciparum* parasites. Expression was normalized across samples by mean centering for each data set. Hierarchical clustering method was applied to the similarity matrix of samples/genes based on Pearson correlation coefficient. Only genes presented in >75% samples of a data set were used for the clustering analysis. a. Heat map of 577 $^{(bl)}$0hr transcriptomes with 4779 representative genes obtained by microarrays. b. Heat map of 459 $^{(tr)}$6hr transcriptomes with 4714 representative genes obtained by microarrays. c. Heat map of 188 $^{(bl)}$0hr transcriptomes with 4305 representative genes obtained by RNA-seq. d. Heat map of 159 $^{(tr)}$6hr transcriptomes with 3923 representative genes obtained by RNA-seq.

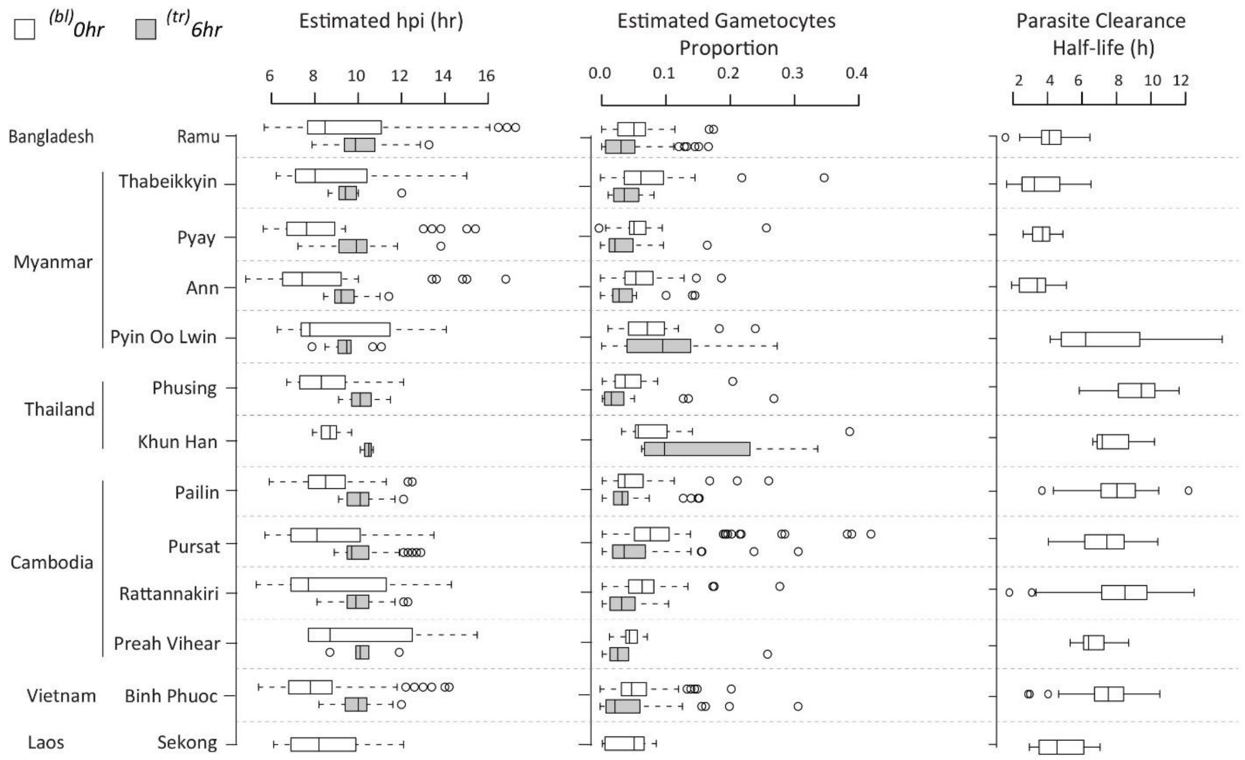


**Supplementary Figure 2**: Distribution of the estimated age/hpi, gametocytes fraction and PC½ across the sampling sites listed by country. The data was shown for all the samples before transcriptome filtering. White boxes represent (bl)0hr samples and grey boxes represent (tr)6hr samples. Each box indicates the interquartile of the data with a median line drawn inside. The whiskers of each box indicate the 1.5 times the interquartile range from the box or the data extremes. The circles indicate outliers beyond that.

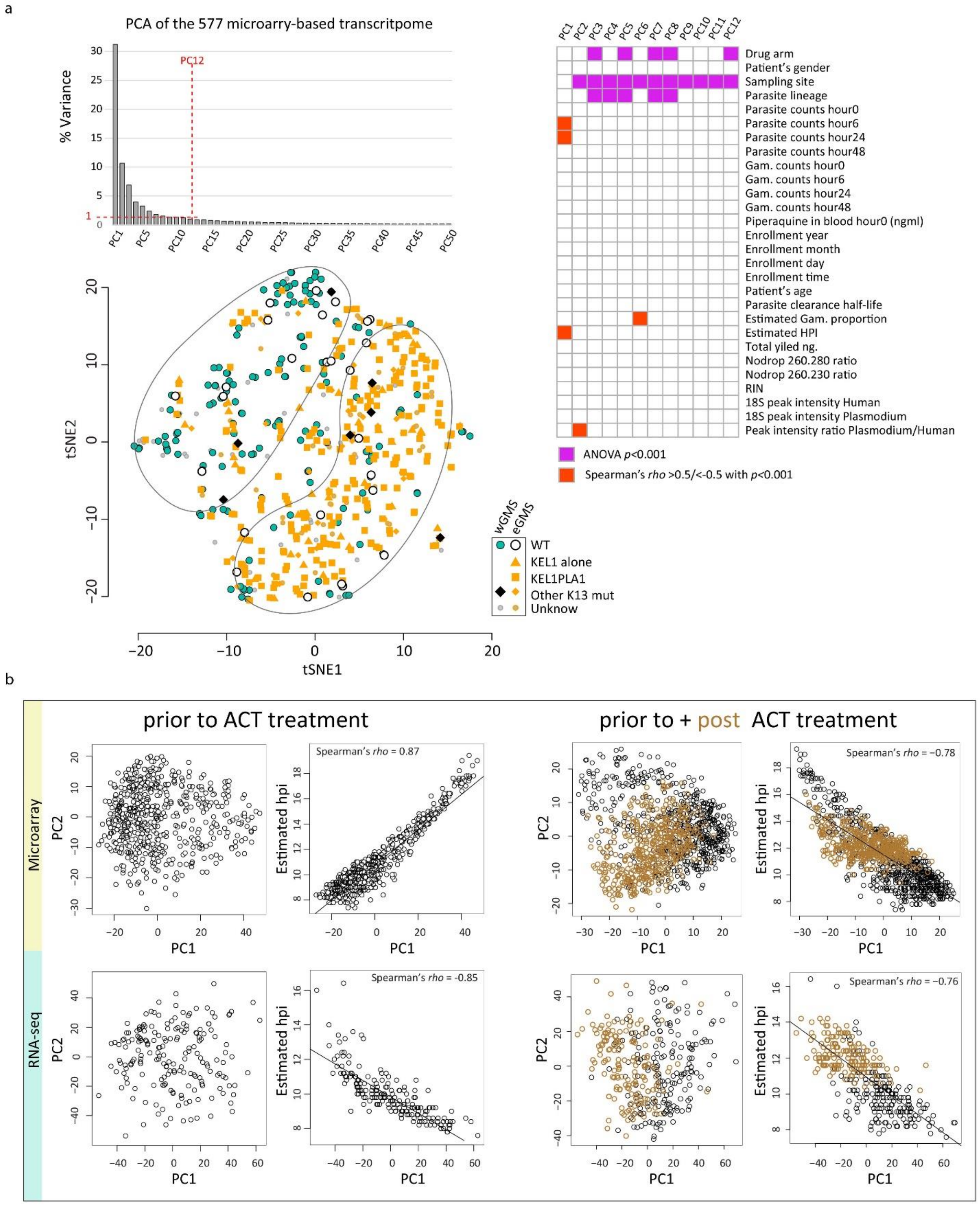
a
PCA of the 577 microarry-based transcritpome
% Variance
PC12
PC1 PC5 PC10 PC15 PC20 PC25 PC30 PC35 PC40 PC45 PC50
PC1 PC2 PC3 PC4 PC5 PC6 PC7 PC8 PC9 PC10 PC11 PC12
Drug arm
Patient's gender
Sampling site
Parasite lineage
Parasite counts hour0
Parasite counts hour6
Parasite counts hour24
Parasite counts hour48
Gam. counts hour0
Gam. counts hour6
Gam. counts hour24
Gam. counts hour48
Piperaquine in blood hour0 (ngml)
Enrollment year
Enrollment month
Enrollment day
Enrollment time
Patient's age
Parasite clearance half-life
Estimated Gam. proportion
Estimated HPI
Total yiled ng.
Nodrop 260.280 ratio
Nodrop 260.230 ratio
RIN
18S peak intensity Human
18S peak intensity Plasmodium
Peak intensity ratio Plasmodium/Human
ANOVA p<0.001
Spearman's rho >0.5/<-0.5 with p<0.001
tSNE2
tSNE1
wGMS eGMS
WT
KEL1 alone
KEL1PLA1
Other K13 mut
Unknow
b
prior to ACT treatment
prior to + post ACT treatment
Microarray
RNA-seq
PC2
PC1
Estimated hpi
Spearman's rho = 0.87
Spearman's rho = -0.85
Spearman's rho = −0.78
Spearman's rho = −0.76

**Supplementary Figure 3**: PCA of the parasite transcriptome. a. Bar plot represents the percent of variance explained by each PC. For the top 12 PCs, each explained >1% of the total transcriptome variance. The relation to clinical or technical factors were tested for each of the 12 PCs. The grid mixing PCs and factors represents the significant association in magenta for the PC-factor pairs passing the threshold of $p<0.001$ with ANOVA test and in red for the PC-factor pairs passing the threshold $p<0.001$ and Spearman's *rho*>0.5. The scatter plot represents the tSNE derived two-dimensional visualization of 577 [(bl)]0hr samples by lineages and geographical regions. b. PC2 and estimated age/hpi were plotted against PC1 individually for each data set ([(bl)]0hr or [(tr)]6hr) by each technology (microarray or RNA-seq).

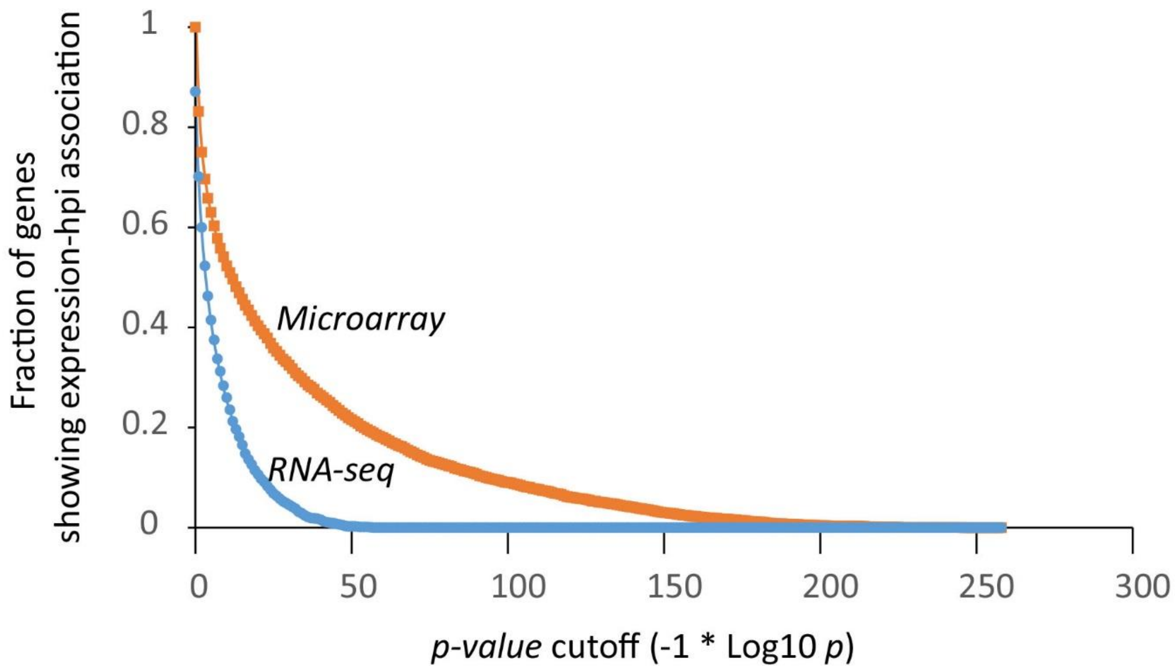


**Supplementary Figure 4:** Expression level correlates to age/hpi for most of the *P. falciparum* genes. P-values were obtained by testing the null hypothesis of no expression change over hpi for each gene using the package gam of R. The curves plotted for Microarray data (orange) and RNA-seq data(blue) to show the gene fractions at each p-value cutoff for defining hpi-associated gene expression.

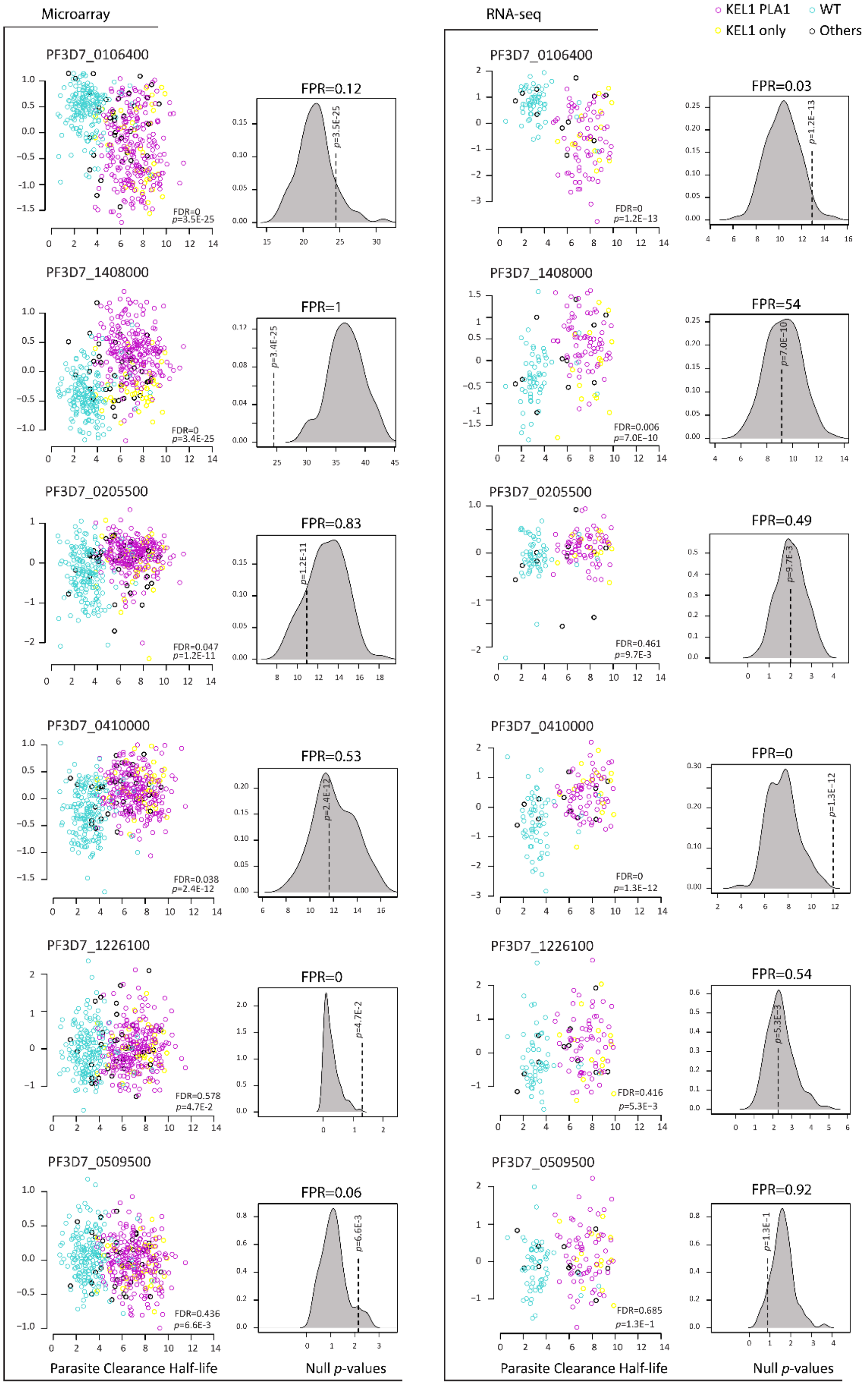

Microarray
RNA-seq
KEL1 PLA1
WT
KEL1 only
Others
PF3D7_0106400
FPR=0.12
FDR=0
p=3.5E-25
FPR=0.03
FDR=0
p=1.2E-13
PF3D7_1408000
FPR=1
FDR=0
p=3.4E-25
FPR=54
FDR=0.006
p=7.0E-10
PF3D7_0205500
FPR=0.83
FDR=0.047
p=1.2E-11
FPR=0.49
FDR=0.461
p=9.7E-3
PF3D7_0410000
FPR=0.53
FDR=0.038
p=2.4E-12
FPR=0
FDR=0
p=1.3E-12
PF3D7_1226100
FPR=0
FDR=0.578
p=4.7E-2
FPR=0.54
FDR=0.416
p=5.3E-3
PF3D7_0509500
FPR=0.06
FDR=0.436
p=6.6E-3
FPR=0.92
FDR=0.685
p=1.3E-1
Parasite Clearance Half-life
Null p-values

**Supplementary Figure 5:** The expression residuals were plotted against the PC½ values with parasite lineage of KEL1PLA1 indicated by purple circles, KEL1 only by yellow, WT by turquoise and others by black for selected genes: TSR2 (PF3D7_0106400), PMII(PF3D7_1408000), PF3D7_0205500 and EVP1(PF3D7_0410000), HAD3(PF3D7_1226100) and PF3D7_1467000 which showed different levels of FDR and FPR. The density plot on the right represents the null *p*-values distribution for FPR calculation based on 100 times permutation of each corresponding gene's resistance status/PC½ values within lineages.

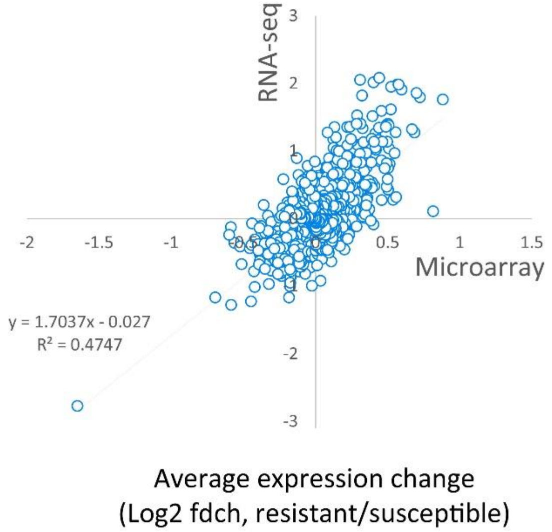


**Supplementary Figure 6:** The average expression change determined by RNA-seq was plotted against that by microarray to show the consistent results of expression-resistance analysis regardless of the methods applied. The average expression change was calculated as the difference between the average transcriptional level (Log2 Ratio) of a gene in the resistant parasites (PC½>5hr) and that in the susceptible parasites (PC½<5hr). Therefore, the average expression change represented here are Log2 fold change of transcriptional level (Log2 fdch). The grey line represents the linear regression with the result indicated in formula on the side.

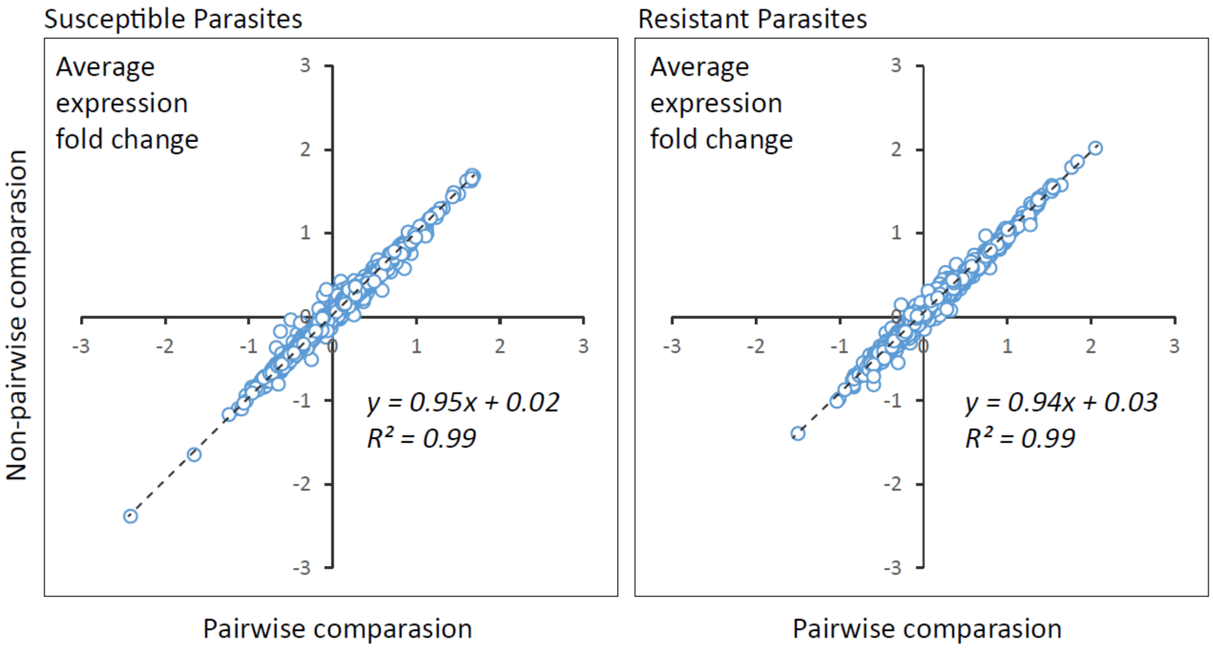


**Supplementary Figure 7**: Average expression fold change of each gene was compared between pairwise analysis and non-pairwise analysis for susceptible parasites and resistant parasites separately. The linear regression was performed for each data set and shown as the dotted line with formula and $R^2$ on the side. The average expression fold change was calculated based on 129 sample pairs for susceptible group and 178 sample pairs for resistant group. Here paired samples are samples collected from the same patient.

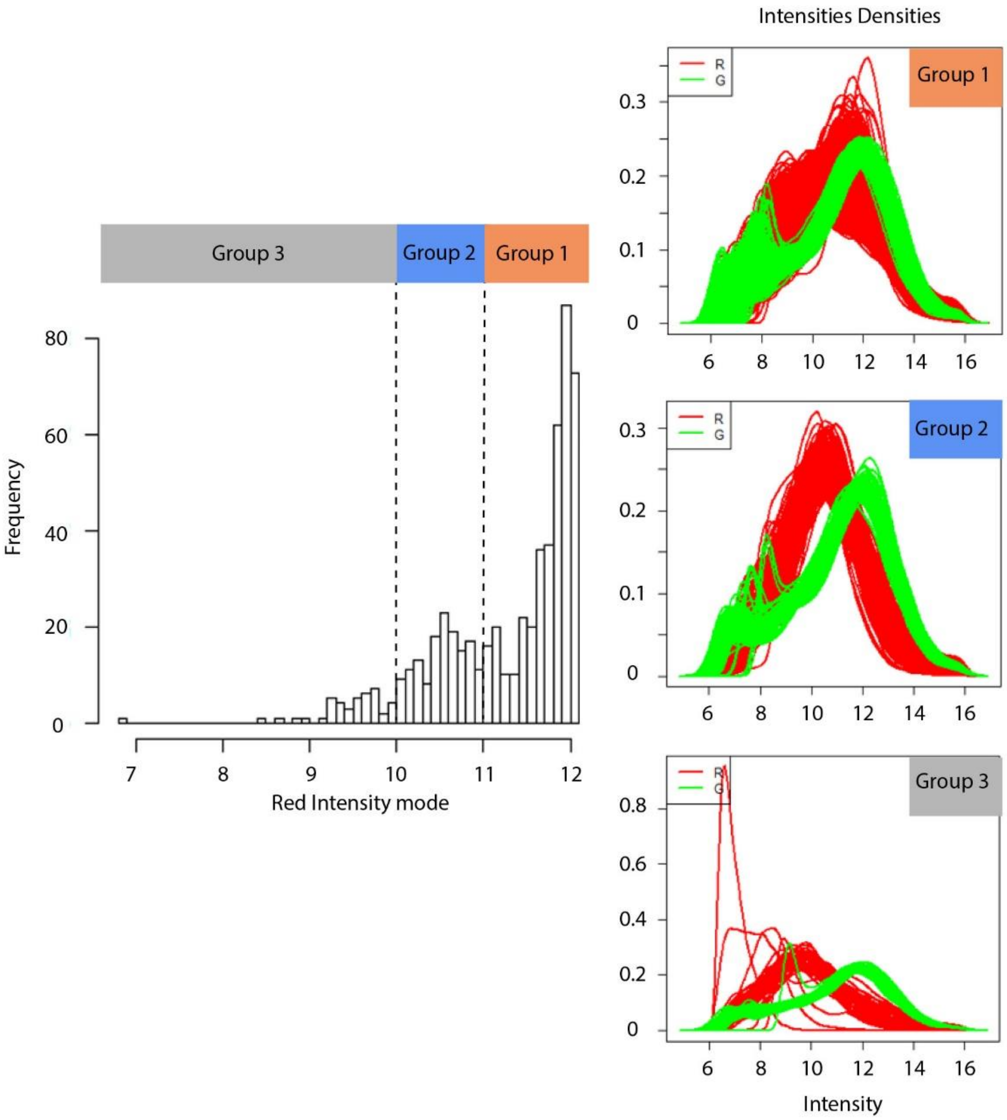


**Supplementary Figure 8**: Defined intensity threshold for microarray-generated transcriptome filtering using the (tr)6hr data set. The histogram on the left represents the overall distribution of red intensity mode values for all the 659 studied arrays. The mode value is the most frequently appearing value in a data set. Here, we estimated it using the intensity value appearing at the biggest peak of corresponding density plot. Arbitrary cutoffs were set at the mode value of 10 and 11 to bin the samples/arrays into 3 groups. The density plot of red intensity (Cy5) and green (Cy3) intensity is drawn for each group on the right. The threshold was set at the mode value of 10 to select samples displaying sufficient signals for the subsequent analysis.
