# Supplementary material for: Artemisinin resistance in the malaria parasite, Plasmodium falciparum, originates from its initial transcriptional response
Source: Commun Biol. 2022 Mar 28;5:274. doi: 10.1038/s42003-022-03215-0 (PMC8960834; doi:10.1038/s42003-022-03215-0)
Supplement: Supplementary file 2 — Editorial Assessment Report [file 42003_2022_3215_MOESM2_ESM.pdf]

Contents of this report

- **Manuscript details**: overview of your manuscript and the editorial team.
- **Review synthesis**: summary of the reviewer reports provided by the editors.
- **Editorial recommendation**: personalized evaluation and recommendation from all 3 journals.
- **Annotated reviewer comments**: the referee reports with comments from the editors.
- **Open research evaluation**: advice for adhering to best reproducibility practices.

About the editorial process

Because you selected the **Nature Portfolio Guided Open Access option**, your manuscript was assessed for suitability in three of our titles publishing high-quality work across the spectrum of genetics research: *Nature Genetics, Nature Communications*, and *Communications Biology*. More information about Guided Open Access can be found here.

Collaborative editorial assessment

Your editorial team discussed the manuscript to determine its suitability for the Nature Portfolio Guided OA pilot. Our assessment of your manuscript takes into account several factors, including whether the work meets the **technical standard** of the Nature Portfolio and whether the findings are of **immediate significance** to the readership of at least one of the participating journals in the Nature Portfolio Guided Open Access genetics cluster.

Peer review

Experts were asked to evaluate the following aspects of your manuscript:

- **Novelty** in comparison to prior publications;
- **Likely audience** of researchers in terms of broad fields of study and size;
- **Potential impact** of the study on the immediate or wider research field;
- **Evidence** for the claims and whether additional experiments or analyses could feasibly strengthen the evidence;
- **Methodological detail** and whether the manuscript is reproducible as written;
- Appropriateness of the literature review.

Editorial evaluation of reviews

Your editorial team discussed the potential suitability of your manuscript for each of the participating journals. They then discussed the revisions necessary in order for the work to be published, keeping each journal's specific editorial criteria in mind.

Journals in the Nature portfolio will support authors wishing to transfer their reviews and (where reviewers agree) the reviewers' identities to journals outside of Springer Nature.

If you have any questions about review portability, please contact our editorial office at guidedoa@nature.com.

Manuscript details

Tracking number	Submission date	Decision date
GUIDEDOA-21-00142	28 May 2021	18 August 2021

**Title**	The mechanism of artemisinin resistance of *Plasmodium falciparum* malaria parasites originates in their initial transcriptional response	**Corresponding author**	Zbynek Bozdech<br>**Affiliation:** Nanyang Technological University
**Preprint information**	There is a preprint of this manuscript posted at bioRxiv	**Peer review type**	Single-blind

Editorial assessment team

**Primary editor**	**Michael Fletcher**<br>**Home Journal:** *Nature Genetics*, ORCID: 0000-0003-1589-7087<br>**Email:** michael.fletcher@nature.com
**Editorial team members**	**Margot Brandt**, *Nature Communications*, ORCID: 0000-0002-9434-794X<br>**George Inglis**, *Communications Biology*, ORCID: 0000-0002-9069-5242
**About your primary editor**	Michael Fletcher studied for his Ph.D. at Cancer Research UK's Cambridge Institute at the University of Cambridge in the laboratory of Bruce Ponder. His project used systems biology approaches to identify the functional mechanism by which FGFR2, a breast cancer risk locus identified using GWAS, exerts its effect. He then moved to Germany and was a postdoctoral fellow in the Molecular Genetics department of Peter Lichter at the German Cancer Research Center in Heidelberg, where he performed bioinformatics analysis to help characterize the epigenomic and master regulator landscapes of adult glioblastoma. He joined the journal in 2020.

Editorial assessment and review synthesis

**Editor's summary and assessment**	This is a transcriptomic study of artemisinin-based combination therapy (ACT) resistance development in *P. falciparum* from the Greater Mekong Region, conducted on samples collected during a clinical trial (TRACII, reported in ref. 2). Mutations in the *PfK13* gene are known to cause resistance, albeit with a still-unclear mechanism; and studies have implicated multiple candidate pathways.<br><br>Here, parasite RNA was collected from patient blood at baseline and 6h post-ACT and profiled using microarrays/RNA-seq. A TWAS (carefully controlling for parasite life cycle stage and other confounders) is used to define an artemisinin resistance-associated transcriptional profile (ARTP) of 156 genes. Further analysis suggests the ARTP correlates with geography and is related to the parasite transcriptional response observed post-ACT in patients.<br><br>Our editorial assessment pre-review was that this manuscript offered some important novelty (the ARTP) and a dataset of useful resource value, addressing a major public health issue (malaria). Our concerns were that the analysis presented is entirely descriptive, and that the degree of advance over past studies in this field (e.g. ref. 39) seemed unclear to us. Nevertheless, we concluded that the importance of the topic and the positive aspects were enough to send this submission out for peer review, most likely for consideration at *Communications Biology*.
**Editorial synthesis of reviews**	Overall, reviewers appreciated the study, describing it as "valuable", "interesting" and "an exceptional resource for understanding resistance". Reviewer #3 (who we asked to examine the computational analysis) points out that the analysis performed is not a TWAS but thinks overall that it is soundly performed and appropriate for the task.<br><br>The malaria experts (Reviewers #1 and #2) both raised the same concern, which should be fully addressed in a revision for Communications Biology: that the extreme population structure of *P. falciparum* has not been properly accounted for and may confound the results. Beyond that, Reviewer #1 also made a number of suggestions for further analyses.

Editorial recommendation

Journal	Decision	Comments
nature genetics	Revision not invited	Due to the unclear advance over the past studies in this field and a lack of novel mechanistic insight into ACT resistance, *Nature Genetics* does not invite a revision.
nature communications	Revision not invited	*Nature Communications* is not inviting a revision due to limited advance over previous malaria transcriptomics manuscripts and a lack of new biological insight.
communications biology	Major Revisions	*Communications Biology* would be interested in a revised manuscript that addresses any potential effects of *P. falciparum* population structure on your analyses (as highlighted by Referees #1-2), and qualifies the description of the underlying methods as a TWAS (per Referee #3).

Next steps

Recommendation Summary

- **Option 1:** Revise for consideration at *Communications Biology*
- **Option 2:** Revise for submission elsewhere

See the previous page for details. As stated, Nature Genetics and Nature Communications can no longer consider the manuscript due to unclear advance over previous work, based on the reviewers' comments.

Revision

To follow our recommendation, please upload the revised manuscript, along with your point-by-point response to the reviewers' reports and editorial advice **using the link provided in the decision letter**.

Revision checklist

- Cover letter, stating to which journal you are submitting
- Revised manuscript
- Point-by-point response to reviews
- Updated **Reporting Summary** and **Editorial Policy Checklist**
- Supplementary materials (if applicable)

Submission elsewhere

To a journal outside of Nature Portfolio

If you choose to submit your revised manuscript to a journal at another publisher, we can share the reviews with another journal outside of the Nature Portfolio if requested. You will need to request that the receiving journal office contacts us at guidedOA@nature.com. We have included editorial guidance below in the reviewer reports and open research evaluation to aid in revising the manuscript for publication elsewhere.

Annotated reviewer reports

The editors have included some additional comments on specific points raised by the reviewers below, to clarify requirements for publication in the recommended journal(s). However, please note that all points should be addressed in a revision, even if an editor has not specifically commented on them.

Reviewer #1

**Reviewer #1**	This reviewer has not chosen to waive anonymity. The reviewer's identity can only be shared with representatives of an established journal editorial office.
**Reviewer #1 expertise**	*P. falciparum* drug resistance, population genetics
**Editor's comments about this review**	This reviewer sounds very positive for the paper and acknowledges its importance for the field. They think that the computational analysis was carefully done and well-explained, but comments that the ARTP may be confounded by geographical stratification caused by founder effects.<br><br>They make a few comments for additional analysis and clarifications, for example trying to distinguish sensitive/resistant parasites using the ARTP.<br><br>Please note that while this reviewer thinks your study is suitable for *Nature Communications* or *Communications Biology*, this is an editorial decision.

Reviewer #1 comments

**Overview**	The manuscript by Zhu, van der Pluijm and Kucharski et al describes a large TWAS for artemisinin response over 577 patients. The study includes samples taken from patients prior to, and after 6 hours of treatment with artemisinin. Patients were recruited across 13 field sites, in 6 countries, representing a major, coordinated effort. The authors tackle several major technical issues within, including the integration of RNAseq with microarray data, non-synchronous samples and variation in hours post infection across samples. There is extreme population stratification across the data, which is directly tackled.<br><br>For a potentially unwieldy study the authors break down the analysis beautifully, initially describing the data, then performing a TWAS for time 0 and relating these findings to the 6 hour responses. To achieve the latter, a panel of genes identified in the second section are used and provide support that the transcriptional signature associated with resistance is present prior to treatment, rather than a response to it.<br><br>Overall, the paper adds further weight to the role cellular stress plays in resistance to artemisinin and provides an exceptional resource for understanding resistance.

Specific comments

#	Reviewer comment	Editorial comment
**1**	I think the article would be suitable for *Nature Communications*, or *Communications Biology*.	As stated above, we appreciate the reviewer's comment but for editorial reasons, *Nature Communications* has decided not to invite a revision.
**2**	A major issue is the confounding due to geographic structure. Most parasites from eastern Greater Mekong Subregion (eGMS) are resistant, and most from wGMS are sensitive. There is no simple way around this, and the authors relay this information clearly in the results. Little to no discussion of this major topic, and how it may impact the results is presented in the discussion.	
**3**	A major feature of the manuscript is the artemisinin response transcriptional phenotype (ARTP) as a marker of resistance. There is a demonstration that these genes overlap with in vitro derived transcriptome studies targeting artemisinin resistance, however I did not see the **panel of genes tested for their ability to distinguish resistant from sensitive parasites**.	We think this would be a useful, additional analysis that would not be unduly hard to perform.
**4**	Conversely, the authors cluster resistant parasite lines using the ART genes and find strong geographic stratification.<br>**Might part of the signal be driven by the founder effects caused by the impact of a huge reduction in the parasite population?** Several of these genes may be linked to artemisinin resistance through this extreme population structure.	We think this is the most important concern raised in these reports; see also Reviewer #2's comments.
**5**	There is strong statistical analysis in the paper, the above comments on population structure notwithstanding. The analysis and data are clearly presented, though the latter would benefit from some better choice of points in some figures (i.e. 1A, S3A). I have a few minor comments on the choices made<br>#1 from Line 194: 477,000 is an odd number for	

	permutations, why was this picked?<br><br>#2 I was confused by the 6-hour analysis. Was this a comparison between paired samples from the same patient to detect up- and down-regulated genes? This would seem like a natural way to treat the data.	

**Reviewer #2**	
**Reviewer #2**	This reviewer has not chosen to waive anonymity. The reviewer's identity can only be shared with representatives of an established journal editorial office.
**Reviewer #2 expertise**	Malaria, genomics, ecology/evolution
**Editor's comments about this review**	This reviewer acknowledges the importance of the work, but overall judges it "interesting but preliminary". Their explanation is that the results may be confounded by population structure, i.e. from geography and/or inbreeding. They point out that the methods applied to correct for this have been used on humans, which do not have the same level of inbreeding as *P. falciparum*.<br><br>While this review is very brief, we thought that this concern – overlapping with Reviewer #1's report – is of clear importance and should be examined in detail in a revision.

Reviewer #2 comments

#	Reviewer comment	Editorial comment
**1**	This is a valuable study that identifies preliminary patterns in the *P. falciparum* transcriptome correlated with phenotypes of ACT tolerance. Whereas the ACT phenotype is clear and well established, a first concern on the patterns emerging is whether they reflect phenotypes not properly accounted for due to geographic differentiation and/or other processes due to the emerging association because of the inbreeding driven by selection rather than causation.<br><br>The authors make reasonable efforts to control for geographic structure using methods developed for humans. However, humans do not have the level of inbreeding that Plasmodium may have. Thus, I think that	The concern about the extent to which the results are caused by population structure (inbreeding) is also shared by reviewer #1.

	these results are interesting but preliminary since it is not clear to what extent they are generated by population structures (not only geography but inbreeding).<br><br>A critical assessment of the results is required, including, the potential spurious associations of genes with the phenotype due to the nature of a life cycle where inbreeding and selection will be linked. So we may end up having two profiles that reflect the characteristics of the strains that were selected for at the moment of the sweep, but such characteristics may not have anything to do with the phenotype of interest. This is not solely a statistical problem, it requires modeling the parasite demography.	

Reviewer #3	
**Reviewer #3**	This reviewer has not chosen to waive anonymity. The reviewer's identity can only be shared with representatives of an established journal editorial office.
**Reviewer #3 expertise**	statistical genetics, genomics
**Editor's comments about this review**	This reviewer – engaged to specifically review the computational analysis – thinks that it is sound and appropriate methodologically, but not a "TWAS". They have a number of minor comments that seem easily addressable.
**Reviewer #3 comments**	
**Overview**	In the manuscript under consideration, the authors have conducted expression profiling to investigate artemisinin resistance of *P. falciparum*. This looks like an interesting study, and it appears that the authors have identified sensible genes in the related pathways.

Specific comments

#	Reviewer comment	Editorial comment
1	Since I was asked by the editors to assess the statistical soundness of the analytic procedure regarding TWAS, I must point out that the authors haven't conducted TWAS at all. What the authors have done is an association analysis between gene expressions and the phenotype, i.e., PC1/2 etc. They haven't used the DNA genotype at all, nor did they use expressions to bridge genotype and phenotype. Nevertheless, the analysis by itself looks fine and the authors may consider removing the term "TWAS" throughout the manuscript.	We recommend removing the term TWAS to more accurately describe the analysis.
2	Why use PC2- 12 for tSNE visualization? Why the PC1 is removed?	This seems clear in your manuscript: PC1 is highly correlated (Spearman's rho = 0.87) with the confounder of parasite life cycle (L162-4).
3	It is unclear on why the authors claim that there are little batch effects based on the fact that little correlation is observed between PC2-12 and known parameters.	
4	Ref 35 is not really the right paper to cite for TWAS. Please try to look at the original papers, e.g., https://www.nature.com/articles/ng.3367 and https://www.nature.com/articles/ng.3506. But as stated above, the work is not related to TWAS; it is just expression analysis.	
5	Remarks to the Author: Impact<br>I am not able to assess the impact to the field of *P. falciparum* study. The statistical method they used looks normal.	
6	Remarks to the Author: Reproducibility<br>The reproducibility in terms of statistical analysis looks good. The authors claimed that the expression data are available at NCBI's Gene Expression Omnibus (GEO) database.	Please ensure that your expression data is uploaded to GEO, so that it can be checked by reviewers. For further details, please see the Open Research Evaluation.

Open research evaluation

Data availability

Data availability statement

Thank you for including a Data Availability statement. However, we noted that you have only indicated that data are available upon request. The data availability statement must make the conditions of access to the “minimum dataset” that are necessary to interpret, verify and extend the research in the article, transparent to readers.

In addition, Nature Portfolio policies include a strong preference for research data to be archived in public repositories. For data types without specific repositories, we recommend that data are deposited in a generalist repository such as figshare or Dryad. More information about our data availability policy can be found here: https://www.nature.com/nature-portfolio/editorial-policies/reporting-standards#availability-of-data

See here for more information about formatting your Data Availability Statement: http://www.springernature.com/gp/authors/research-data-policy/data-availability-statements/12330880

Mandatory data deposition

For your RNA sequencing data, submission to a community-endorsed, public repository is mandatory for publication in a Nature Portfolio journal and is best practice for publication in any venue. Accession numbers must be provided in the paper. Examples of appropriate public repositories are listed below:

- Gene Expression Omnibus (Microarray or RNA sequencing data)
- Sequence Read Archive (high-throughput sequence data)
- The European Nucleotide Archive (ENA)

For more information on mandatory data deposition policies at the Nature Portfolio, please visit http://www.nature.com/authors/policies/availability.html#data

For a list of approved repositories for each mandatory data type, please visit https://www.springernature.com/gp/authors/research-data-policy/repositories/12327124

For your gene expression microarray data, submission to a community-endorsed, public repository is mandatory for publication in a Nature Portfolio journal and is best practice for publication in any venue. Accession numbers must be provided in the paper. Examples of appropriate public repositories are listed below:

- ArrayExpress
- Gene Expression Omnibus (GEO)
- Genomic Expression Archive

For more information on mandatory data deposition policies at the Nature Portfolio, please visit http://www.nature.com/authors/policies/availability.html#data

For a list of approved repositories for each mandatory data type, please visit https://www.springernature.com/gp/authors/research-data-policy/repositories/12327124

Please ensure that datasets deposited in public repositories are now publicly accessible, and that accession codes or DOI are provided in the "Data Availability" section. As long as these datasets are not public, we cannot proceed with the acceptance of your paper. For data that have been obtained from publicly available sources, please provide a URL and the specific data product name in the data availability statement. Data with a DOI should be further cited in the methods reference section.

Ethics

Because your study includes human participants, confirmation that all relevant ethical regulations were followed is needed, and that informed consent was obtained. This must be stated in the Methods section, including the name of the board and institution that approved the study protocol.

Reporting & reproducibility

Nature Portfolio journals allow unlimited space for Methods. The Methods must contain sufficient detail such that the work could be repeated. It is preferable that all key methods be included in the main manuscript, rather than in the Supplementary Information. Please avoid use of “as described previously” or similar, and instead detail the specific methods used with appropriate attribution.

We encourage you to share your step-by-step experimental protocols on a protocol sharing platform of their choice. The Nature Portfolio’s Protocol Exchange is a free-to-use and open resource for protocols; protocols deposited in Protocol Exchange are citable and can be linked from the published article.

More details can be found at www.nature.com/protocolexchange/about

Please state in the legends how many times each experiment was repeated independently with similar results. This is needed for all experiments, but is particularly important wherever results from representative experiments (such as micrographs) are shown. If space in the legends is limiting, this information can be included in a section titled "Statistics and Reproducibility" in the methods section.

Statistics and data presentation

When choosing a color scheme please consider how it will display in black and white (if printed), and to users with color blindness. Please consider distinguishing data series using line patterns rather than colors, or using optimized color palettes such as those found at https://www.nature.com/articles/nmeth.1618

The use of colored axes and labels should be avoided. Please avoid the use of red/green color contrasts, as these may be difficult to interpret for colorblind readers.

The quality of some of the figures appears to be quite low. If possible, we suggest replacing these with higher-resolution images.

Data presentation: Please ensure that data presented in a plot, chart or other visual representation format shows data distribution clearly (e.g. dot plots, box-and-whisker plots). When using bar charts, please overlay the corresponding data points (as dot plots) whenever possible and always for $n \leq 10$. (Please see the following editorial for the rationale behind this request and an example https://www.nature.com/articles/s41551-017-0079).

Statistics: Wherever statistics have been derived (e.g. error bars, box plots, statistical significance) the legend needs to provide and define the n number (i.e. the sample size used to derive statistics) as a precise value (not a range), using the wording "n=X biologically independent samples/animals/cells/independent experiments/n= X cells examined over Y independent experiments" etc. as applicable

Legends requiring revision:

1. Please note that this information is missing in the legend of supplementary figure 2

Statistics such as error bars, significance and p values cannot be derived from n<3 and must be removed from all such cases.

We strongly discourage deriving statistics from technical replicates, unless there is a clear scientific justification for why providing this information is important. Conflating technical and biological variability, e.g., by pooling technically replicates samples across independent experiments is strongly discouraged. (For examples of expected description of statistics in figure legends, please see the following https://www.nature.com/articles/s41467-019-11636-5 or https://www.nature.com/articles/s41467-019-11510-4).

All error bars need to be defined in the legends (e.g. SD, SEM) together with a measure of centre (e.g. mean, median). For example, the legends should state something along the lines of "Data are presented as mean values +/- SEM" as appropriate. All box plots need to be defined in the legends in terms of minima, maxima, centre, bounds of box and whiskers and percentile.

Legends requiring revision:

1. Please note that the box plots need to be defined in terms of minima, maxima, centre, bounds of box and whiskers and percentile in the legend of supplementary figure 2

The figure legends must indicate the statistical test used. Where appropriate, please indicate in the figure legends whether the statistical tests were one-sided or two-sided and whether adjustments were made for multiple comparisons. For null hypothesis testing, please indicate the test statistic (e.g. F, t, r) with confidence intervals, effect sizes, degrees of freedom and P values noted. Please provide the test results (e.g. P values) as exact values whenever possible and with confidence intervals noted.

Legends requiring revision:

1. Please indicate the statistical test used for data analysis and where appropriate, please specify whether it was one-sided or two-sided and whether adjustments were made for multiple comparisons, in the legends of figure 1b; table 1; supplementary figures 4; 5 and supplementary data 2.

2. Please note that the information on whether the statistical test used was one-sided or two-sided, where appropriate, is missing in the legends of figures 2a, b; 3d and supplementary figure 3a.

3. Please note that the exact p value should be provided, when possible, in the legends of figures 2a, b; 3d and supplementary figure 3a.

Other notes

We have included as an attachment to the decision letter a version of your Reporting Summary with a few notes. This is mainly for your information, but we hope it is helpful when preparing your revised manuscript. If you decide to resubmit the manuscript for further consideration, please be sure to include an updated Reporting Summary.
