# Supplementary material for: Artemisinin resistance in the malaria parasite, Plasmodium falciparum, originates from its initial transcriptional response
Source: Commun Biol. 2022 Mar 28;5:274. doi: 10.1038/s42003-022-03215-0 (PMC8960834; doi:10.1038/s42003-022-03215-0)
Supplement: Supplementary file 3 — List of Supplementary Information [file 42003_2022_3215_MOESM3_ESM.docx]

Supplementary Data 1: Parasite age and gametocyte proportion estimation
Supplementary Data 2: Expression markers of resistance and drug response genes
