# Supplementary material for: Artemisinin resistance in the malaria parasite, Plasmodium falciparum, originates from its initial transcriptional response
Source: Commun Biol. 2022 Mar 28;5:274. doi: 10.1038/s42003-022-03215-0 (PMC8960834; doi:10.1038/s42003-022-03215-0)
Supplement: Supplementary file 6 — Reporting Summary [file 42003_2022_3215_MOESM6_ESM.pdf]

nature portfolio

Corresponding author(s): Zbynek Bozdech

Last updated by author(s): Feb 14, 2022

Reporting Summary

Nature Portfolio wishes to improve the reproducibility of the work that we publish. This form provides structure for consistency and transparency in reporting. For further information on Nature Portfolio policies, see our Editorial Policies and the Editorial Policy Checklist.

Statistics

For all statistical analyses, confirm that the following items are present in the figure legend, table legend, main text, or Methods section.

n/a	Confirmed	
☐	☒	The exact sample size (*n*) for each experimental group/condition, given as a discrete number and unit of measurement
☐	☒	A statement on whether measurements were taken from distinct samples or whether the same sample was measured repeatedly
☐	☒	The statistical test(s) used AND whether they are one- or two-sided<br>*Only common tests should be described solely by name; describe more complex techniques in the Methods section.*
☐	☒	A description of all covariates tested
☐	☒	A description of any assumptions or corrections, such as tests of normality and adjustment for multiple comparisons
☐	☒	A full description of the statistical parameters including central tendency (e.g. means) or other basic estimates (e.g. regression coefficient) AND variation (e.g. standard deviation) or associated estimates of uncertainty (e.g. confidence intervals)
☐	☒	For null hypothesis testing, the test statistic (e.g. *F*, *t*, *r*) with confidence intervals, effect sizes, degrees of freedom and *P* value noted<br>*Give P values as exact values whenever suitable.*
☒	☐	For Bayesian analysis, information on the choice of priors and Markov chain Monte Carlo settings
☒	☐	For hierarchical and complex designs, identification of the appropriate level for tests and full reporting of outcomes
☐	☒	Estimates of effect sizes (e.g. Cohen's *d*, Pearson's *r*), indicating how they were calculated

Our web collection on statistics for biologists contains articles on many of the points above.

Software and code

Policy information about availability of computer code

Data collection	The raw transcriptome data was generated by customized microarray chip using commercially available hybridization platform or Illumina HiSeq4000 platform. The microarray generated gpr files were processed using Limma package of R. And the RNA-seq generated fastq files were processed using Trim Galore, Hisat2, Samtools and BEDtools.
Data analysis	R packages were used for respective data analysis: stats package for linear regression, Anova test, T test and clustering method. Plinks was applied to the population analysis.

For manuscripts utilizing custom algorithms or software that are central to the research but not yet described in published literature, software must be made available to editors and reviewers. We strongly encourage code deposition in a community repository (e.g. GitHub). See the Nature Portfolio guidelines for submitting code & software for further information.

Data

Policy information about availability of data

All manuscripts must include a data availability statement. This statement should provide the following information, where applicable:

- Accession codes, unique identifiers, or web links for publicly available datasets
- A description of any restrictions on data availability
- For clinical datasets or third party data, please ensure that the statement adheres to our policy

All data is available in the main text or the supplementary materials or from the corresponding author upon request. All transcriptome data used in this study are available at NCBI’s Gene Expression Omnibus (GEO) database with the series accession number GSE149735 for microarray and GSE169520 for RNA-seq.

Field-specific reporting

Please select the one below that is the best fit for your research. If you are not sure, read the appropriate sections before making your selection.

☒ Life sciences ☐ Behavioural & social sciences ☐ Ecological, evolutionary & environmental sciences

For a reference copy of the document with all sections, see nature.com/documents/nr-reporting-summary-flat.pdf

Life sciences study design

All studies must disclose on these points even when the disclosure is negative.

Sample size	In the TRACII study, 680 malaria-infected patients' samples were collected from 6 countries and 13 sites across Southeast Asia Greater Mekong Region. With each patient, parasite samples were collected from the venous blood at admission to the clinic (Hour 0, 0hr) and 6 hours after their respective treatment (Hour 6, 6hr).
Data exclusions	We only considered samples with high quality data for this analyses and removed those samples with less representative transcriptome or low signal intensities/sequencing quality. At last, 577 samples pre-treatment and 459 samples post-treatment passed our criteria for microarray method. And 188 samples pre-treatment and 159 samples post-treatment passed our criteria for RNA-seq.
Replication	Due to the limitation of sample collection with the filed parasites, we do not have biological sample replicates. we provides technical replicates as we could for samples(188 pre-treatment and 159 post-treatment) using two transcriptome measurement platforms, RNA-seq and microarray. We compared and intergated the results.
Randomization	FDR was estimated accordingly to control the false positive. And, the half-life status were randomized within parasite lineages and FPR was estimated to reduce the confounding effects of lineages and resistance.
Blinding	Not applicable. The TRACII project aims to study the mechanism behind the artemisinin resistance.

Reporting for specific materials, systems and methods

We require information from authors about some types of materials, experimental systems and methods used in many studies. Here, indicate whether each material, system or method listed is relevant to your study. If you are not sure if a list item applies to your research, read the appropriate section before selecting a response.

Materials & experimental systems

n/a	Involved in the study	
☒	☐	Antibodies
☒	☐	Eukaryotic cell lines
☒	☐	Palaeontology and archaeology
☒	☐	Animals and other organisms
☒	☐	Human research participants
☒	☐	Clinical data
☒	☐	Dual use research of concern

Methods

n/a	Involved in the study	
☒	☐	ChIP-seq
☒	☐	Flow cytometry
☒	☐	MRI-based neuroimaging
